# The novel hsa-miR-12528 regulates tumourigenesis and metastasis through hypo-phosphorylation of AKT cascade by targeting IGF-1R in human lung cancer

**DOI:** 10.1038/s41419-018-0535-8

**Published:** 2018-05-01

**Authors:** Seong Ho Jeon, Jung Ki Yoo, Chang Min Kim, Eun Su Lim, So Jeong Lee, Ji Min Lee, Seung-Hun Oh, Jin Kyeoung Kim

**Affiliations:** 1 Department of Pharmacy, College of Pharmacy, CHA University, 689 Sampyeong-dong, Bundang-gu, Seongnam-si, Gyeonggi-do 463-400 Republic of Korea; 2 Department of Neurology, CHA Bundang Medical Center, CHA University, Seongnam-si, Gyeonggi-do 463-954 Republic of Korea

## Abstract

Lung cancer cases are increasing yearly; however, few novel therapeutic strategies for treating this disease have been developed. Here the dysregulation between microRNAs and oncogenes or tumour-suppressor genes forms a close connection-loop to the development or progression in human lung carcinogenesis. That is, the relationship between microRNAs and carcinogenic mechanism may find the critical clue to improve the treatment efficacy. Accordingly, we identified and characterised a novel microRNA, hsa-miR-12528, in A549 cells. The miR-12528 expression was aberrantly downregulated in cancer cell lines and in the patient tissues derived from human non-small cell lung cancer. In addition, we found that miR-12528 post-transcriptionally controls the translation of the insulin-like growth factor 1 receptor (IGF-1R) gene by directly targeting the 3′-untranslated region of IGF-1R mRNA. Notably, the IGF-1R gene is elevated in the majority of cancers and may be an attractive therapeutic target for anticancer therapy because elevated IGF-1R mediates the signalling amplification of a major oncogenic pathway in neoplasia. In A549 cells, miR-12528 overexpression epigenetically altered the downstream phosphorylation of the primary IGF-1R networks, negatively regulated proliferation, apoptosis and migratory activity, and consequently inhibited tumourigenesis and metastasis in vivo. Therefore, our discovery of hsa-miR-12528 may be able to be applied to the development of molecular-target therapeutic strategies and diagnosis-specific biomarkers for human lung cancer.

## Introduction

Lung cancer, which is worldwide public health problem, is of critical concern because its mortality rate is increasing yearly. Epithelial lung cancer is commonly classified into two types: small cell lung cancer (SCLC) and non-small cell lung cancer (NSCLC). Approximately 80% of lung cancer patients are classified as NSCLC types, and 40–60% of NSCLC cases are classified as adenocarcinoma^1^. Previous studies suggested that certain miRNAs can delay NSCLC development and progression. For instance, miR-29b^1^ and -9500^2^ dysregulation in NSCLC can repress cellular proliferation and metastasis by silencing oncogenic pathway, such as *ID1* and *Akt*.

MicroRNAs (miRNAs) are endogenous small non-coding RNAs. Generally, mature miRNAs are 17–25 nucleotides (nt) and are derived from a precursor of 70–100 nt, which forms a secondary hairpin structure. During biogenesis, the majority of miRNAs require editing by *Drosha*, *Dicer* ribonuclease and *DGCR8* auxiliary protein in the nucleus and the cytoplasm^3,4^.

The miRNA duplex liberated by dicing is matured by an interaction with the RNA-induced silencing complex or protein–RNA complexes that are formed from RNA-binding factors, such as the argonaute proteins^5^. The mature miRNA binds complementarily to the 3′-untranslated region (UTR) of its target messenger RNA (mRNA), cleaves the target mRNA and/or suppresses translational levels. Therefore, miRNAs can silence target genes^6^.

Several genetic factors such as pro-oncogenes contribute to the biological development or progression of malignant lung cancer. Here insulin-like growth factor 1 receptor (IGF-1R) is considered to be an attractive factor for molecular-targeted therapy. The IGF-1R consists of heterotetramer, which has two alpha subunits in the extracellular membrane and two beta subunits in the intracellular membrane. Insulin growth factor 1 (IGF-1), IGF-2 and insulin bind the alpha subunit of IGF-1R, and IGF-1 has a high affinity for IGF-1R. Beta subunits or domains mediate signal transduction cascades^7^. Tyrosine phosphorylation of IGF-1R upon extracellular ligand binding induces the activation of insulin receptor substrate 1 that can provide sufficient binding of initial effector-associated tyrosine phosphorylation genes with an SH2-domain, such as *PI3K*, *Shc* and *Grb2*, through pleckstrin homology and phosphotyrosine-binding domains^8,9^. Namely, IGF-1R internalisation induces the hyperphosphorylation of PI3K/Akt/mTOR signalling pathway and then can promote cellular proliferation, metastasis and tumourigenesis^10^.

In previous studies, several miRNAs, such as miR-7^11^, miR-122^12^, miR-139^13^ and miR-375^14^ have shown therapeutic potential that can delay the progression and development of certain human cancers by epigenetically interfering of primary signalling by targeting IGF-1R. In particular, miR-140^15^ and miR-195^16^ decrease tumour growth and metastasis through proliferative delay or arrest by targeting IGF-1R in NSCLC. Therefore, these miRNAs for anti-IGF-1R may serve as biological therapeutic agents and early diagnosis.

In our study, we cloned small RNAs in lung cancer cells to investigate their potential use in NSCLC therapy or diagnosis. Here we identified a novel miRNA, hsa-miR-12528, that was analysed with regard to its secondary structure and expression patterns in cell lines/tissues related to NSCLC. We also assessed the target genes of miR-12528 and performed a functional study in NSCLC. Our results demonstrate that hsa-miR-12528 can regulate tumourigenesis and metastasis of certain NSCLC cells in vitro and in vivo.

## Results

### Basic information, expression profiling and influence on the novel hsa-miR-12528 in lung cancer

**Fig. 1:**
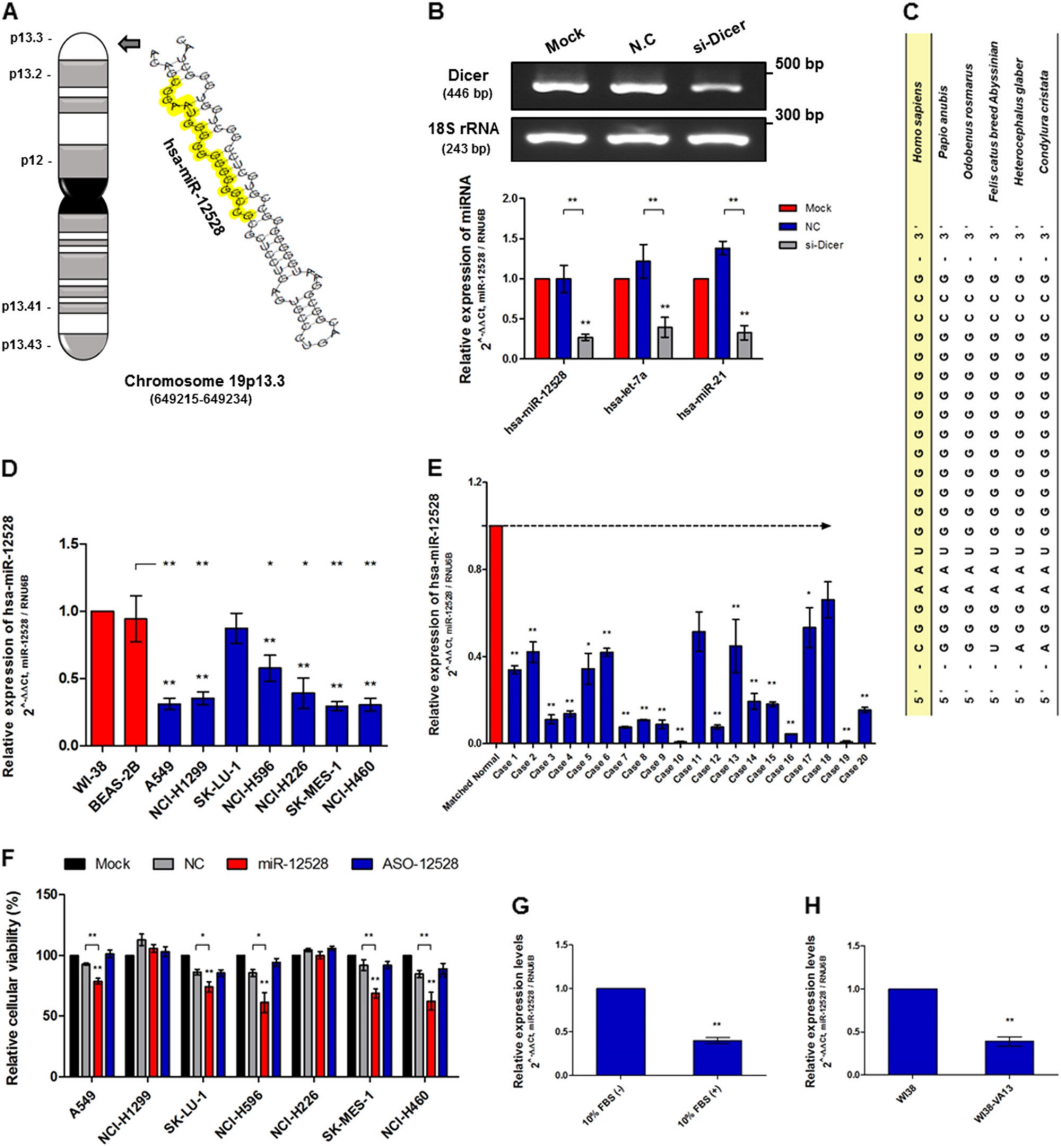
Basic information, expression profiling and influence on the novel hsa-miR-12528 in lung cancer. Identification and cloning of the miR-12528 from lung cancer cells. The assumed secondary folding structure of miR-12528. Human genomic sequences were found using the RNAfold web-tool. The marked location is a mature miR-12528 sequence. The miR-12528 is located on chromosome 19p.13.3 (649215-649234) (**a**). Maturation of miR-12528 is dependent on the Dicer pathway, as shown via *Dicer*-knockdown and an miScript miRNA assay (**b**). Mature miR-12528 is conserved at a high rate in other species. These results were determined using the NCBI BLAST tool (**c**). The miR-12528 expression was assessed in both 7 NSCLC cell lines (**d**) and 20 pairs of NSCLC patient tissues (**e**) compared with normal WI-38 and BEAS-2B cells, and matched normal tissues. When classified histologically, cases 1–10 are adenocarcinoma type and cases 11–20 are squamous cell carcinoma type (**e**). The proliferative activity in 7 NSCLC cells was assessed using a XTT reagent 48 h post transfection with 100 nM mimics (**f**). The miR-12528 expression levels were observed using qRT-PCR between absence and presence of FBS stimulation (**g**) and between diploid normal WI-38 and immortalised WI-38 VA13 (**h**). These expression profiling data were normalised to RNU6B and performed in triplicate using an miScript assay on independent samples (bars, mean ± S.E.M.; *p < 0.05 and **p < 0.01)

We identified and cloned hsa-miR-12528 in A549 cells. The secondary structure for miR-12528 was predicted using the RNAfold web programme. The miR-12528 mature sequence was identified (5′-CGGAAUGGGGGGGGGGGCCG-3′) using T-vector cloning and sequencing. The transcription site is located on the intergenic region between the *FGF22* and *PSTL3* genes on chromosome 19p13.3 (649215-649234) (Fig. 1a). The miR-12528 expression was similarly reduced in A549 cells with the knockdown of the endogenous *Dicer* gene compared with known miRNAs, miR-21 and let-7a (Fig. 1b). The miR-12528 sequence is conserved at a high rate of 95% in other species. These bioinformatics data were generated using National Council for Biotechnology Information (NCBI) Basic Local Alignment Search Tool (BLAST) and the Ensemble genome browser (Fig. 1c). To investigate its potential biological functions, we assessed miR-12528 expression levels. The results showed that miR-12528 expression levels were downregulated in almost NSCLC cells and in 20 NSCLC tissue samples compared with WI-38 (normal lung fibroblast), BAE-2B (normal lung/bronchial epithelial cells) cells and matched normal lung tissues (Fig. 1d, e). Moreover, when overexpression of miR-12528 was induced in these NSCLC, cellular proliferation was decreased with a sensitivity of 20–50% compared with vehicle- and ASO-12528-transfected cells.  However, the proliferation of NCI-H1299 and -H226 cells was not significantly different despite overexpression of miR-12528 (Fig. 1f). Continually, miR-12528 expression levels were assessed between A549 cells stimulated with foetal bovine serum (FBS) and unstimulated A549 cells, and also between WI-38 and WI-38 VA13 cells to determine relationship in the growth of cells. The results showed that miR-12528 expression was downregulated in growing A549 cells and immortalised WI-38 VA13 cells (Fig. 1g, h).

### Relationship between the IGF-1R gene and hsa-miR-12528 in A549 cells

**Fig. 2:**
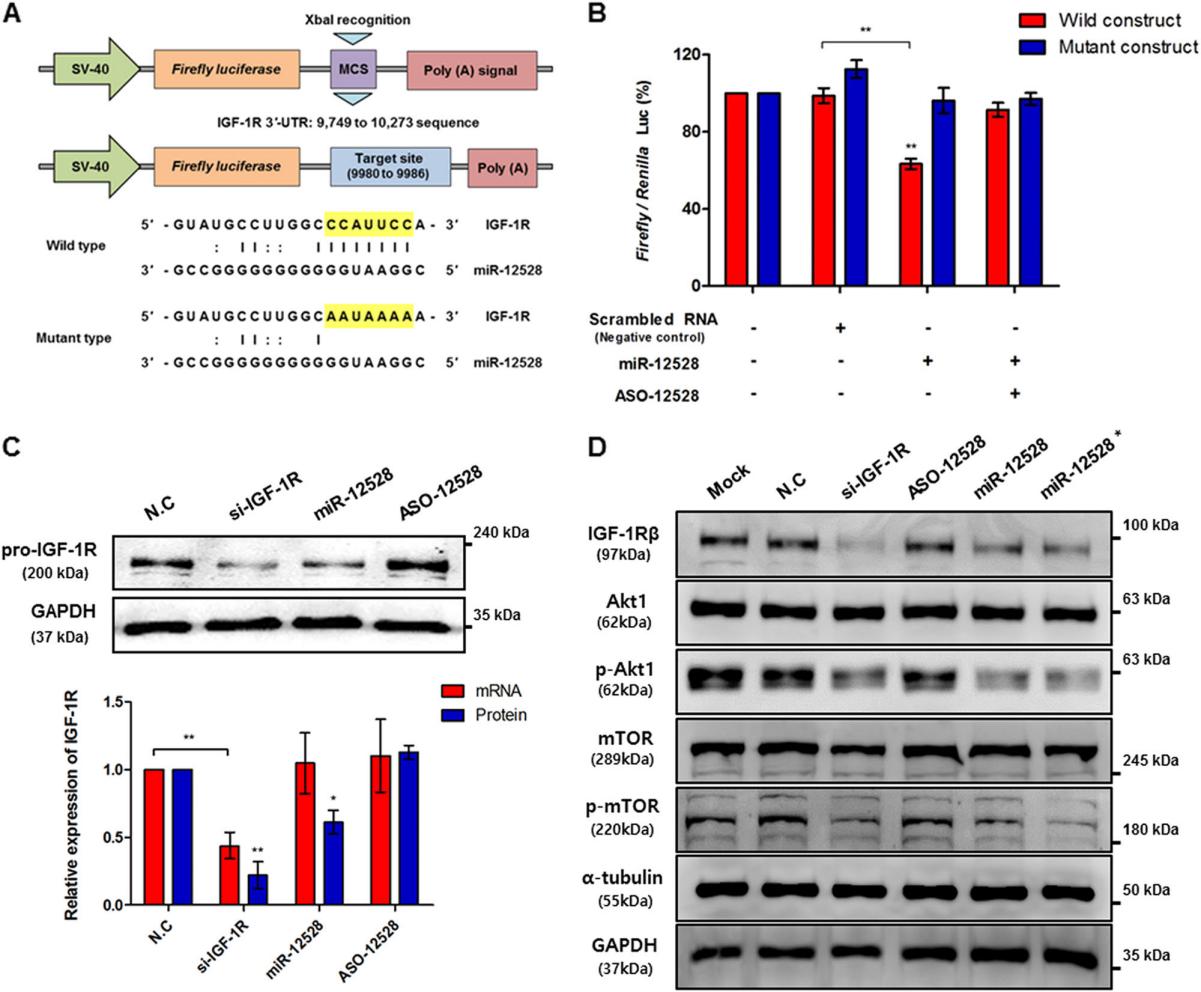
Relationship between the IGF-1R gene and hsa-miR-12528 in A549 cells. The target validation of miR-12528. The pGL3-plasmid was recombined by inserting the 3′-UTR of IGF-1R. The binding sequence of miR-12528 is marked in yellow fills (top; wild type, WT), and was replaced with a mismatched sequence using site-directed mutagenesis (bottom; mutant, MT) (**a**). Luciferase activity of WT and MT was analysed by co-transfecting the recombinant pGL3 construction, pRL-TK and miRNA mimics, and normalised to pRL-TK; *Renilla* luciferase (**b**). The expression levels between mRNA and protein of IGF-1R gene were assessed using quantitative RT-PCR and western blot analysis and normalised to 18S rRNA or GAPDH. Western blot analysis was semi-quantified using NIH ImageJ programme (**c**). The signalling alteration of the IGF-1R-downstream pathway was analysed using a western blot analysis 48 h post transfection (mimic conc.; 50 nM and *; 100 nM) (**d**) (bars, mean ± S.E.M; *p < 0.05 and **p < 0.01)

To test the potentiality of direct target of miR-12528, pGL3 wild-type (WT) plasmid was used to insert the 3′-UTR 9749–10 273 sequences of IGF-1R mRNA containing the assumed recognition regions. In addition, the assumed recognition elements for the miR-12528 seed sequence within WT plasmid were redesigned as a mutant plasmid (MT) by replacing the elements with mismatched bases (Fig. 2a). As a result, WT luciferase activity was decreased, whereas MT luciferase activity was not significantly different in miR-12528-transfected cells compared with the control cells. Furthermore, when co-transfected miR-12528 and its complementary ASO-12528, WT luciferase were not decreased (Fig. 2b). Consecutively, in A549 cells, miR-12528 overexpression decreased the endogenous levels of IGF-1R protein but did not affect the endogenous levels of IGF-1R mRNA, unlike si-IGF-1R action. In addition, the effect of miR-12528 on regulating the IGF-1R was validated in not only the A549 cells but also the eight human-derived cell lines; NCI-H226; -H596; -H460; SK-MES-1 (lung carcinoma); HEK293T (embryonic kidney); Hep G2 (liver hepatocellular carcinoma); MCF7 (breast carcinoma); and HeLa (cervix carcinoma) (Figure S1, Fig. 2c). To understand the biological capacity, the effects of miR-12528 were analysed on the primary IGF-1R signalling cascade. In contrast to NC and ASO-12528-transfected cells, the phosphorylated protein levels of the Akt and mTOR were relatively decreased in the A549 cells where the protein levels of IGF-1R were reduced by si-IGF-1R and miR-12528 (Fig. 2d).

### The anticancer effect of hsa-miR-12528 in cell cycle and apoptosis pathway

**Fig. 3:**
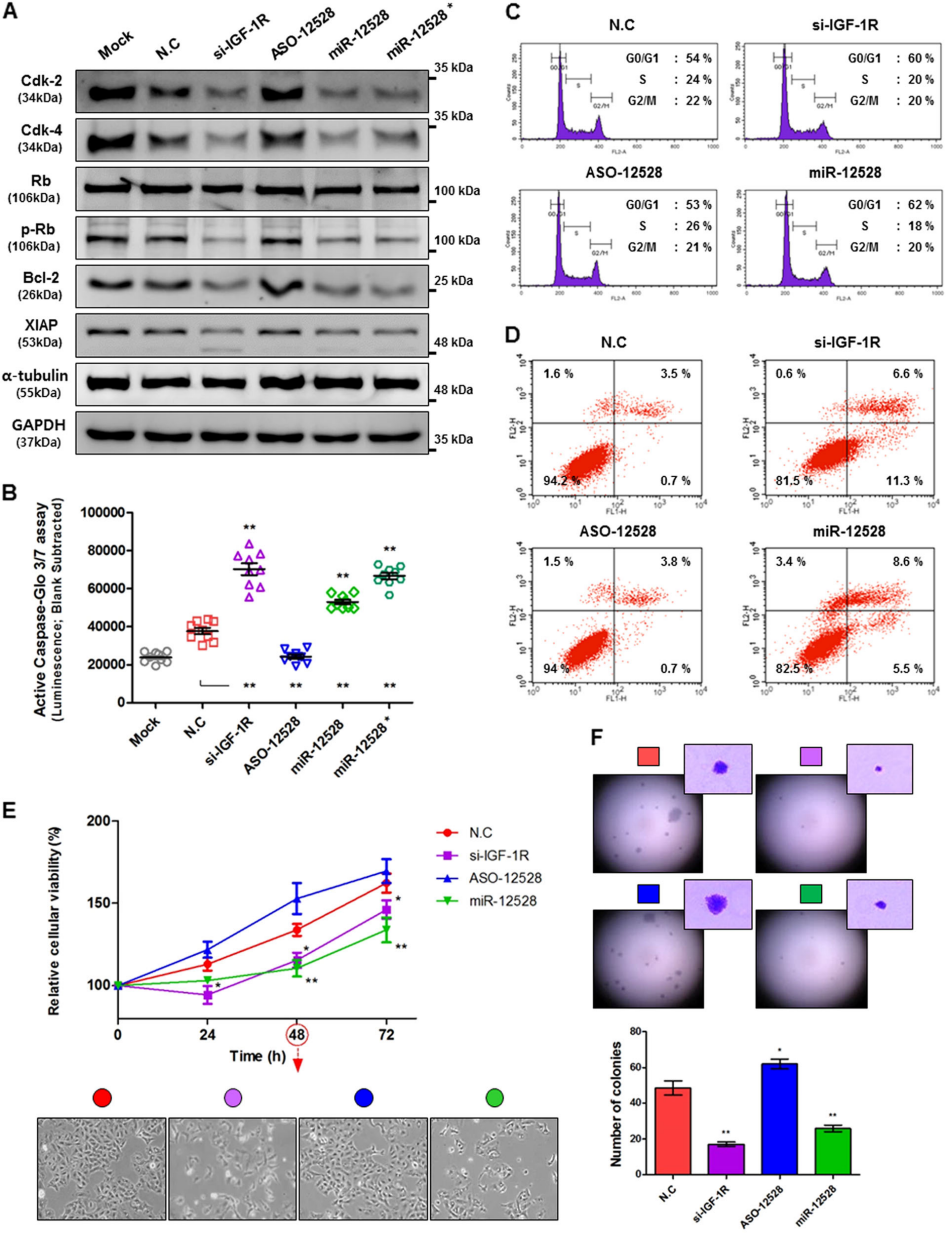
The anticancer effect of hsa-miR-12528 in cell cycle and apoptosis pathway. The signalling alteration and effect for miR-12528 on cell cycle and apoptosis pathway. Forty-eight hours post transfection, Cdk-2, Cdk-4, Rb and pRb proteins in cell cycle pathway with Bcl-2 and XIAP, anti-apoptotic proteins, were analysed using a western blotting (**a**). Caspase-3/-7 activity, apoptotic execution factors, assessed using a Caspase-Glo^®^ 3/7 reagent (mimic conc.; 50 nM and *; 100 nM) (**b**). FACS analysis was performed using flow cytometry in A549 cells fixed or permeabilized 48 h post transfection. Cell cycle distribution was determined according to DNA stained with PI (**c**). Apoptotic cell death was sorted using Annexin V-FITC and PI staining. Annexin V-FITC (Annexin V^+^/PI^−^) indicates that the distribution of apoptosis in the inner leaflet of the phospholipid bilayer of the plasma membrane is translocated to the outer leaflet where the plasma membrane is intact. Annexin V^+^/PI^+^ indicates the distribution of late apoptotic cell death (**d**). Influence of miR-12528 on cell viability in vitro. In short-term proliferative rates, A549 cells were determined to be time-dependent via a XTT assay after transfection with 100 nM miRNA mimics. Cell morphology was imaged at 48 h (×100 magnification) (**e**). After mimic transfection, A549 cell-derived colonies were formed in soft agar for 3 weeks at 37 °C with 5% CO_2_. Graphical presentation shows the representative single colonies at high-power views (×100 magnification) and the numbers of colony-forming units (**f**). The experiments were performed in triplicate for independent samples (bars, mean ± S.E.M.; *p < 0.05 and **p < 0.01)

To examine the anticancer effects of miR-12528 on the hypo-phosphorylation of the Akt/mTOR pathway, we assessed cell cycle, apoptosis and cell viability in miR-12528- or si-IGF-1R-overexpressed cells compared with NC and ASO-12528-transfected A549 cells. The miR-12528 overexpression relatively decreased Cdk-2, Cdk-4 and phosphorylated Rb expression levels in cell cycle pathway. Additionally, Bcl-2 and XIAP, anti-apoptotic factors, were reduced, whereas caspase-3 and -7 activity, execution factors of apoptosis, were increased in both miR-12528- and si-IGF-1R-transfected A549 cells (Fig. 3a, b). In fluorescence-activated cell sorting (FACS) analysis, the G1-phase population was relatively increased and simultaneously, the S- or G2-/M-phase population was relatively decreased. Furthermore, apoptotic cells stained with Annexin V-fluorescein isothiocyanate (FITC)/propidium iodide (PI) were relatively increased in both miR-12528- and si-IGF-1R-transfected A549 cells (Fig. 3c, d, Figure S2). Continually, the knockdown of endogenous miR-12528 in WI-38, normal cells, increased the expression of endogenous IGF-1R and simultaneously cellular proliferation (Figure S3), whereas the miR-12528 and si-IGF-1R transfection in A549 cells inhibited colony-forming ability/size during the long term as well as cell proliferation in a short term (Fig. 3e, f).

### The capacity of hsa-miR-12528 during tumourigenesis in an in vivo model

**Fig. 4:**
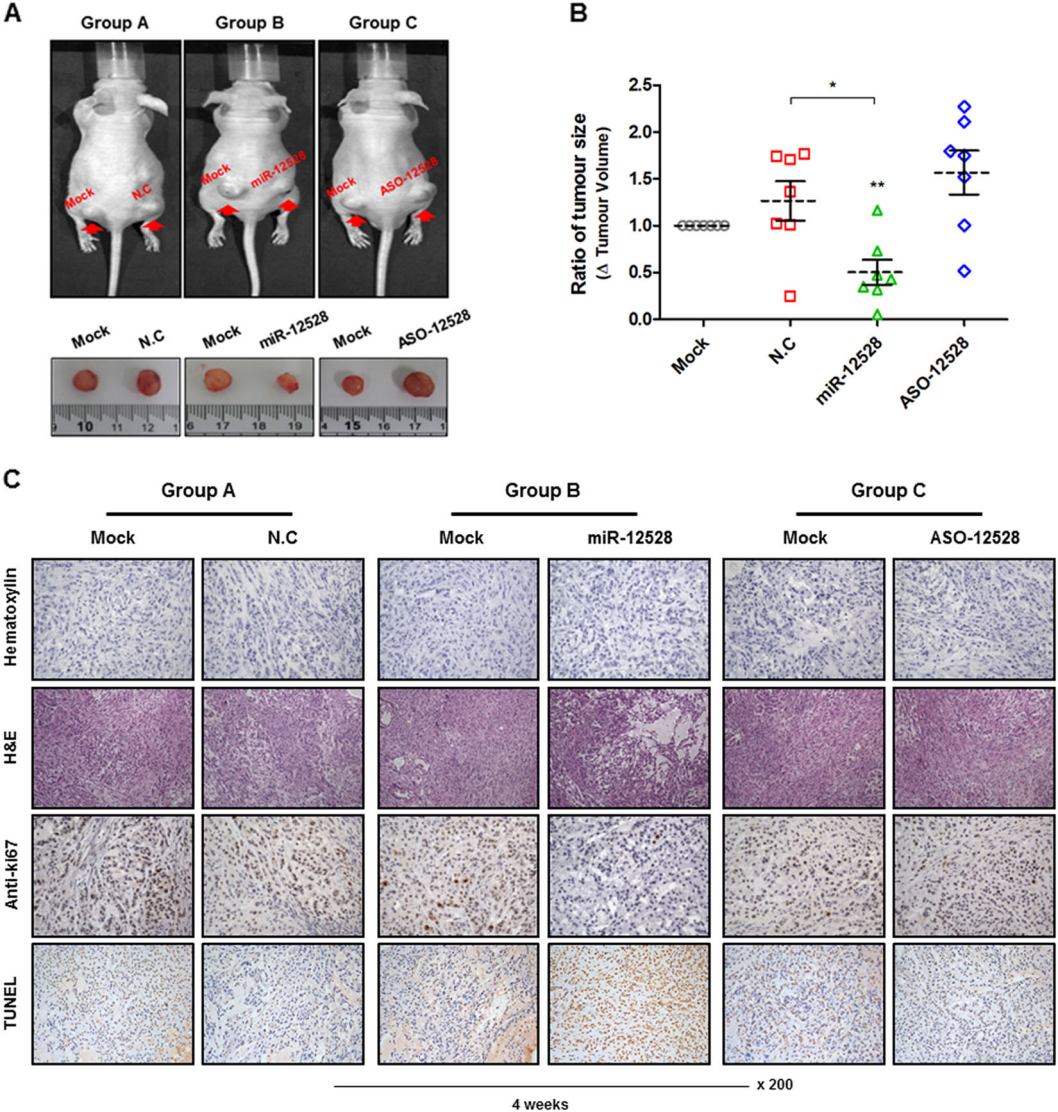
The capacity of hsa-miR-12528 during tumourigenesis in an in vivo model. Influence of miR-12528 during tumourigenesis in vivo. A549 cells were subcutaneously injected in equal amounts into both flanks in an individual mouse. After stabilisation for 5 weeks, the left tumours were only injected with PBS, whereas the right tumours were injected with either NC, miR-12528 or ASO-12528 mimics twice weekly. After a total of 4 weeks from the time of mimic injection, 7 mice per group were sacrificed (a total of 21 mice) and the excised tumour mass was imaged and measured (**a**). The rations of tumour volumes indicate that the right tumours injected with NC, miR-12528 or ASO-12528 mimics were normalised to a left tumour (MOCK) within each individual mouse (**b**). The tumour sections were expressed with H&E staining, Ki67-IHC and TUNEL assay. The H&E staining result shows the histological morphology of tumour tissues. Positive results for Ki67 and TUNEL, markers of proliferation and apoptosis, were indicated by brown nuclear staining (**c**)

During tumourigenesis in subcutaneous xenograft models, we examined the biological effect of miR-12528. The tumour xenograft models were stabilised for approximately 5 weeks after injection of A549 cells to generate equal tumourigenesis. After 5 weeks, although the generated tumour mass in 5 mice was independent on the equal conditions due to variations of growth phenotype in mice, it was similar in both the right and left tumours within each individual mouse. Therefore, to minimise the error values for the discrepancy of tumour masses, the right tumour, which was injected with miRNA mimics, was normalised to a left tumour injected only with phosphate-buffered saline (PBS) (Figure S4). After 4 weeks of miRNA mimic injections, 7 mice per experimental group (a total of 21 mice) were sacrificed and the excised tumour mass was recorded or imaged (Fig. 4a). The volumes of excised NC, miR-12528 and ASO-12528 tumour masses were normalised to a mock-treated tumour (the left position) within each individual mouse (Fig. 4b). Resultingly, the tumour masses exposed to miR-12528 mimics were relatively decreased after 4 weeks from the time of the periodic injection compared with the tumour masses exposed to NC and ASO-12528 mimics (Fig. 4a, b). In a histological analysis, tumour tissue sections showed that the periodic injection of miR-12528 relatively promoted cell death. Moreover, Ki67 expression positively correlated with TUNEL labelling, such that the stained Ki67 proliferation factor-positive cells were relatively decreased, whereas the labelled TUNEL-positive cells were relatively increased in the tumour sections exposed to miR-12528 for 4 weeks compared with the NC and ASO-12528 tumour tissue sections (Fig. 4c).

### Influence of hsa-miR-12528 on migratory activity in vitro and in vivo

**Fig. 5:**
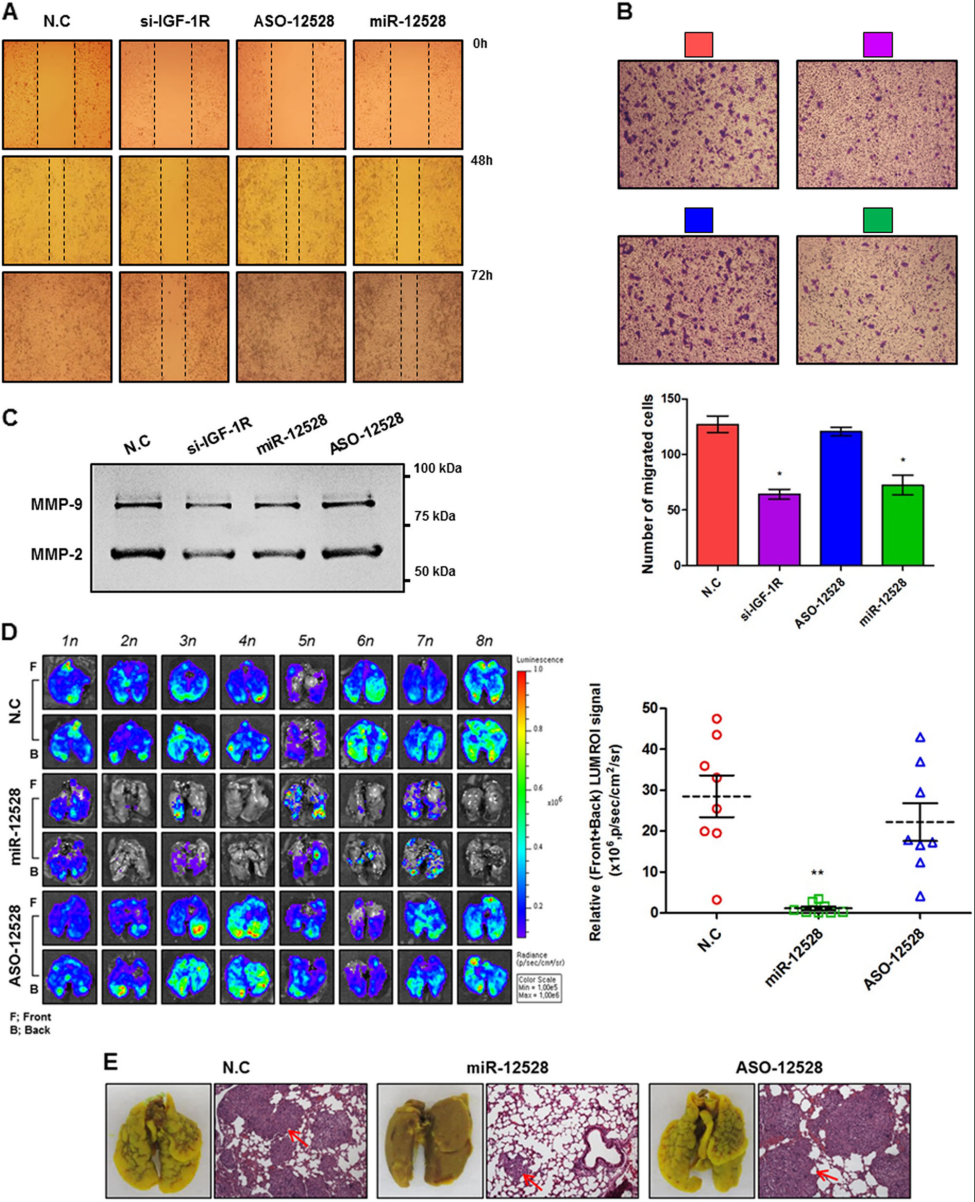
Influence of hsa-miR-12528 on migratory activity in vitro and in vivo. Post transfection, the delayed cell motility was captured in a time-dependent manner in scratched A549 cells (**a**). Cell migration or invasion was measured after 48 h post transfection to allow for the permeabilization of the Trans-well membrane (**b**). Proteolytic enzyme activity of the secreted MMP-2 and -9 was analysed in the cultured soup for 48 h post transfection (**c**). The lung metastatic models that were injected with the miRNA mimic-transfected Fluc-stable A549 cells into the tail vein were grown for 5 weeks (*n* = 8 mice/group). After 5 weeks, the expressed Fluc signals visually show the degree of metastatic spread to lung, and Fluc signal intensity was quantified by ROI gates (**d**). Nodules generated on the lung surface (left) and histological staining (H&E) of lung tissues (right) visually show a distinct difference between each group (**e**) (bars, mean ± S.E.M.; *p < 0.05 and **p < 0.01)

Cellular metastasis is an important feature in lung cancer progression. In our study, the transfection of miR-12528 or si-IGF-1R relatively suppressed cellular motility or approximately 30–40% of migratory activity (Fig. 5a, b) and relatively reduced the enzymatic activity of matrix metalloproteinase-2 (MMP-2) and MMP-9 in A549 cells compared with NC and ASO-12528-transfected cells (Fig. 5c). For more accurate analysis, we analysed the degree of lung metastatic spread in bioluminescence in vivo imaging models. In mice sacrificed after 5 weeks tail vein injection of cells transfected with NC, miR-12528 and ASO-12528 mimic, luciferase activity of lung tissues exposed to miR-12528 mimic was weakly expressed, whereas the luciferase activity of lung tissues exposed to NC or ASO-12528 mimic was strongly expressed (Fig. 5d). Furthermore, histological haematoxylin and eosin (H&E) staining showed that the frequency of nodules on the lung surface between NC or ASO-12528 and miR-12528 groups was distinctly different (Fig. 5e).

## Discussion

The hsa-miR-12528 miRNA is a novel miRNA of 20 nt that we cloned from A549 cells. This novel miRNA is derived from a secondary structure hairpin fold of approximate 80 nt and is conserved in other species. The miR-12528 encoding is located on chromosome 19p13.3 and transcribed from an intergenic region. In biogenesis, most miRNAs are matured by dicing from pre-miRNAs^3–5^. Likewise, we identified that the maturation of miR-12528 depended on the Dicer pathway for miRNA biogenesis because the expression of miR-12528 was downregulated in Dicer-silenced A549 cells (Fig. 1a–c). Consecutively, we analysed the expression pattern of miR-12528 in several environments to investigate the potential biological functions of miR-12528. Previous studies reported that miR-140^15^, miR-145^17^, miR-195^16^ and miR-9500^2^ are downregulated in NSCLC-related biospecimens. Dysregulation of these miRNAs can regulate NSCLC development through the cellular proliferative arrest, survival and metastasis. In our findings, transcriptional levels of miR-12528 were aberrantly downregulated in mostly NSCLC-derived cell lines and in 20 pairs of patient tissues (Fig. 1d, e, Figure S5B). Especially, in these NSCLC cells, we further confirmed that miR-12528 overexpression suppresses the proliferation of several NSCLCs (Fig. 1f). Interestingly, transcriptional levels of miR-12528 were differed between growing and quiescent cells, as absence or presence environment of serum in A549 cells and diploid normal WI-38 and immortalised WI-38 VA13 (Fig. 1g, h). The discrepancies of expression of miR-12528 suggest that its expression may be involved in the cell development process or proliferative senescence and thus could play an important role in NSCLC carcinogenesis.

To investigate the anticancer effects of miR-12528 in molecular levels, targets of the miR-12528 were predicted and evaluated. Behind efforts, we identified that the seed region of miR-12528 directly target the sequences (9980–9986) recognised within the 3′-UTR of the IGF-1R, such as the ratio of Fluc/Rluc activity was significantly decreased with the WT in contrast to the MT and miR-12528-neutralised WT. Furthermore, the overexpression of miR-12528 affects protein synthesis of the IGF-1R in the several human-derived cell lines, including A549 cells (Figure S1, Fig. 2a–c), while neutralising the endogenous miR-12528 in WI-38, normal cells, promotes the expression of IGF-1R (Figure S3). Therefore, these results indicate that the miR-12528 can suppress translational levels by directly targeting recognition elements within the 3′-UTR to post-transcriptionally control IGF-1R genes.

In the relationship between miR-12528 and IGF-1R expression, the deficiency of endogenous IGF-1R expression can also affect the transcript of miR-12528. However, their endogenous expression in NSCLC patient tissues seems not to directly interact with each other despite suggesting a crucial crosstalk between them (Figure S5, Fig. 1e). As mentioned in Fig. 1g, h, this result is thought that miR-12528 expression may be bypassed and increased due to a promotion of growing to quiescent cell transition or proliferative arrest during IGF-1R silencing. On the other hands, the epigenetic abnormalities of miR-12528 in NSCLC may be governed by another signal circulation including IGF-1R, thus sustaining carcinogenic progression. Accordingly, we have additionally found the assumed four genes (*EGFR*, *SRC*, *NRAS* and *IKBKβ*) that allow the alignment on the seed region of miR-12528 with the target mRNA, and then their potential targetability was assessed through luciferase reporter gene assay. However, the result revealed that there was not significantly different except for the IGF-1R (Figures S1E, F). Therefore, although we could not identify other targets of the miR-12528 in this study, they may be contributed to the major loop between aberrant IGF-1R and miR-12528 expression during oncogenesis.

While miR-12528 post-transcriptionally silenced the IGF-1R, presumably its influence was expected to have an anticancer effect, due to an induction of the G1-/S-phase transition arrest in the cell cycle and simultaneously the programmed cell death by intervening primary kinase phosphorylation cascade such as Akt/mTOR pathway in IGF-1R network. (Figs. 2d and 3a–d, Figure S2). Hypo-phosphorylation of Akt/mTOR pathway can result in the G1- to S-phase transition delay during cell division cycle by the signalling intervention of cell cycle-downstream pathway^18^. Moreover, the reduction of Akt/mTOR signalling by suppression of IGF-1R executes the programmed cell death pathway, such as a decrease of anti-apoptotic proteins and an increase of pro-apoptotic proteins, and results in apoptosis^19,20^. As a result of these signalling alteration, previous studies reported that small interfering RNA targeting to IGF-1R gene inhibits invasion and metastasis in in vivo models injected with A549 cells^21^. In particular, miR-122^12^, miR-139^13^ and miR-140^15^ targeting to IGF-1R gene play important key roles during tumourigenesis or metastatic spread in an in vivo model of various malignant cells.

During the growth of A549 cells, we confirmed that the actions of miR-12528 inhibit the cellular proliferation and the long-term colony-forming ability by epigenetically interfering in such signalling transductions (Fig. 3e, f). In addition, the knockdown of endogenous miR-12528 in WI-38 cells elevates the proliferation in a time-dependent manner (Figure S3). In in vivo results, the results of histological assays such as IHC and TUNEL labelling showed that in tumour tissue sections exposed to a mimic of miR-12528, proliferation or apoptosis of tumour cells was negatively regulated (Fig. 4). Therefore, these anti-proliferative effects of miR-12528 could delay the development or progression of tumourigenesis by the induction of G1-phase cell cycle arrest and apoptosis.

Another concern in the majority of metastatic NSCLC patients, which have low survival or high mortality rates, is focused on local recurrence or distant metastasis. In our findings, we demonstrated that miR-12528 can suppress the cellular motility and migratory activity of A594 cells (Fig. 5a, b). What’s more, injection of miR-12528 remarkably decreased the frequency of nodules generated during the metastatic spread to the lung. Absolutely, the intensity of Fluc activity expressed on the lung surface and histological H&E staining revealed that the degree of metastatic spread to the lung showed a distinct difference between miR-12528 groups and other control groups (Figure S6, Fig. 5d, e).

The process of metastasis or invasion is associated with enzymatic degradation of the extracellular matrix (ECM), which is very important for metastatic cancer. Generally, destruction of the ECM requires the activation of proteolytic enzymes such as MMP-2 and MMP-9^22^, and activation of MMP-2 and MMP-9 promotes cancer invasion and metastasis^23^. Previous studies have confirmed that IGF-1R-mediated downstream signalling can modulate protein secretory expression of MMP-2 and MMP-9^24^. Furthermore, silencing of IGF-1R was shown to inhibit enzymatic activities, such as MMP-2 and MMP-9, in human metastatic cancers^13,21^. As mentioned above, these results suggest that miR-12528 could suppress the destruction of the ECM, due to reduced enzymatic activities of MMP-2 and MMP-9, and ultimately inhibits lung metastasis and invasion (Fig. 5c).

**Fig. 6:**
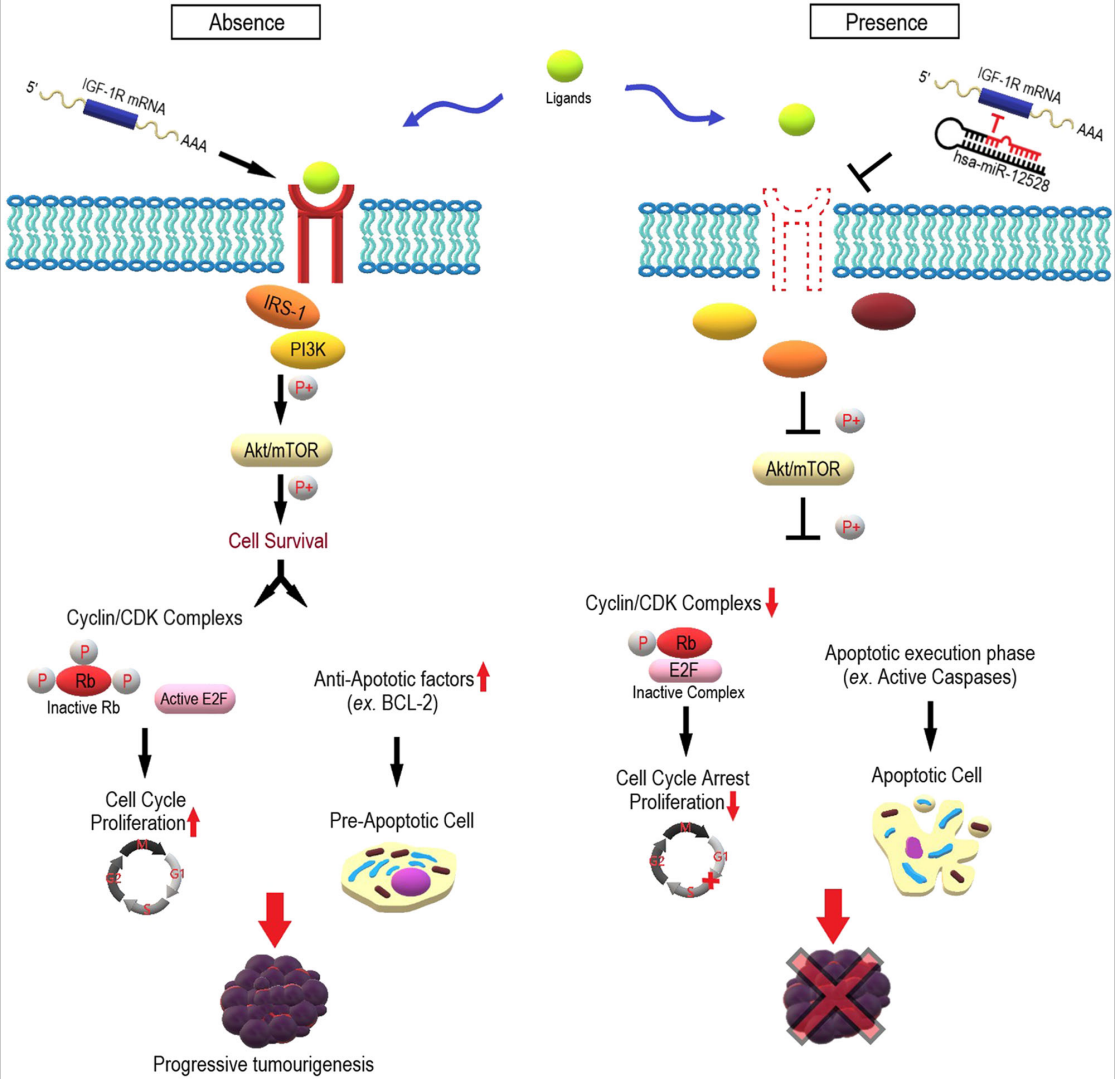
Signalling alteration of IGF-1R networks in the absence and presence of the hsa-miR-12528. The dynamic signalling graphics model based on the presence and absence of miR-12528. The upregulated IGF-1R in NSCLC mediates the interaction of ligands such as IGF-1. Upon internalization between the extracellular ligand and receptor, IGF-1R networks mediate the hyperphosphorylation of the Akt/mTOR, which subsequently leads to the development or progression of NSCLC. In the presence of miR-12528, blocking IGF-1R translation leads to a reduction of the interaction between IGF-1R and its ligand via the lack of the ligand-binding regions, and induces hypo-phosphorylation. Consequently, miR-12528 can negatively regulate NSCLC progression by modulating the cell cycle and apoptotic programmed cell death

In summary, we identified and cloned a novel miRNA, hsa-miR-12528, which is remarkably downregulated in human NSCLC. The miR-12528 overexpression can induce the epigenetic silencing of translation through the post-transcriptional control of the IGF-1R gene. As a result, presence of miR-12528 can induce an epigenetic alteration of such intracellular signalling by blocking the translational levels of the IGF-1R gene. Consequently, miR-12528 can suppress tumour growth and metastasis during tumourigenesis and metastatic spread to the lung (Fig. 6). However, the anticancer effect of miR-12528 alone was restrictive in certain NSCLC, and thus our interest in molecular-target therapy is still considered. Therefore, further analyses, such as the validation of target genes and their networks for miR-12528 in molecular levels, could lead to novel approaches in cancer therapy. Furthermore, the miR-12528 can be used as an IGF-1R regulator, but it should be also considered to work together as a synergistic inhibitor with miRNAs that modulate same target pathway to achieve the dominant inhibition of target. Understanding the biological effects of miR-12528 might lead to the development of a novel anticancer therapeutic target and more specific biomarkers for human NSCLC.

## Materials and Methods

### MiRNA cloning and sequencing

Small RNAs <200 nt in length were isolated using the mirVana RNA isolation Kit (Ambion, Foster City, CA, USA) in A549 cells. The isolated small RNAs were cloned into vectors using the ^Dyna^Express-miRNA cloning Kit (BioDynamics Laboratory Inc., Tokyo, Japan); The small RNAs were separated with a 15% denaturing PAGE gel. The 15- to 30-nt RNAs were dephosphorylated using alkaline phosphatase, and the small RNAs were ligated to 3′ linkers (these products were blocked at the 3’-end, which prevented re-circularisation at the 5′-end). The linker-ligated RNAs were separated with a 15% denaturing gel, and 36- to 46-nt RNAs were extracted, purified and synthesised into cDNA using reverse transcriptase. The PCR products were cloned into the T-A vector (Promega, Madison, WI, USA) and sequenced (Macrogen Inc., Seoul, Korea). The detailed protocol is described previously^25^.

### Bioinformatic analysis

Small RNA sequence was assessed for the presence of miRNAs. The location of miRNAs in the human genome and other characteristics were determined using the NCBI BLAST analysis. The stem loop or secondary structure of miRNAs was examined using the RNAfold webserver.

### Human tissue collection

The tissue samples (biospecimens) and its information for lung cancer were provided by the Korea University Guro Hospital of National Biobank, a member of the National Biobank of Korea. Tissue samples analysed in this study were performed in the 20 NSCLC tissues, regardless of the age and sex. Normal tissue samples, which matched with tumour tissues, were derived from an individual 20 NSCLC patient. The information regarding the biospecimens is noted in the Supplementary Table 1.

### Cell culture

Cell lines (WI-38, WI-38 VA13, BEAS-2B, A549, NCI-H1299, SK-LU-1, NCI-H596, NCI-H226, SK-MES-1 and NCI-H460) were maintained and cultured in culture medium supplemented with 10% FBS (Welgene, Deagu, Korea), 1% penicillin (100 U/ml, Welgene) and streptomycin (100 µg/ml, Welgene) at 37 °C with 5% CO_2_. The information and details on cell culture are summarised in the Supplementary Experimental procedures, Supplementary Information.

### Molecular studies

A549 cells were transfected or overexpressed by a miRNA or siRNA mimic (Genolution Pharmaceuticals Inc., Seoul, Korea)-Lipofectamine 2000 (Invitrogen, Carlsbad, CA, USA) mixture, according to the manufacturers’ protocol. Total RNA was isolated using the TRIzol reagent (Qiagen, Hilden, Germany) as previously described^26^. cDNA was synthesised using the reverse transcription Kit (Invitrogen) and the miScript II RT Kit (Qiagen), according to the manufacturers’ protocol. The results of miRNA or gene profiling were analysed using the quantitative reverse transcription-PCR (RT-PCR) analysis (Bio-Rad, Hercules, CA, USA) and expressed as 2(−delta (delta) threshold cycle) values (2^−∆∆(C)T^) with reference previously described^27^. Proteins were extracted using a PRO-PREP™ (iNtRON, Seoungnam, Korea) lysis buffer that was supplemented with phosphatase inhibitor cocktail (Thermo Scientific, Rockford, IL, USA), fractionated by sodium dodecyl sulphate-polyacrylamide gel electrophoresis (SDS-PAGE) and transferred onto polyvinylidene fluoride membranes (pore size, 0.45 µm, GE Healthcare Life Sciences, Piscataway, NJ, USA). The details for molecular studies performed in this study, such as cDNA synthesis, miRNA mimic design, transfection, miScript miRNA assay, RT-PCR analysis and western blot analysis are described in the Supplementary Experimental procedures, Supplementary Information.

### Target gene-dual luciferase reporter gene assay

Approximately 500 nt of IGF-1R 3′-UTR containing the predicted miR-12528-binding site were inserted into the pGL3 WT plasmid (Promega). Mutations of the binding sites were generated using a Muta-Site-Directed Mutagenesis Kit (iNtRON, Seoungnam, Korea) and a control vector was used as pRL-TK (Promega) of *Renilla* luciferase. The pmirGLO constructs (Promega) were also redesigned by inserting the 3′-UTR sequences of the IGF-1R, EGFR, SRC, NRAS and IKBKB that are complementary matched with the seed region of miR-12528. A549 cells (5 × 10^4^ cells/well) were plated in 24-well plates. The next day, pGL3 or pmirGLO construction (100 ng), pRL-TK (50 ng) and miRNA mimic (100 nM) were co-transfected into each well. Luciferase activity was measured using a Dual-Luciferase Reporter Assay System Kit (Promega) and analyser VICTOR^3^ (PerkinElmer Inc., Foster City, CA, USA). All experiments were performed in triplicate in independent samples.

### Cell survival assay

Proliferation assay was assessed using a XTT test solution (Roche, Mannheim, Germany) according to the manufacturers’ protocols. And the transfected cells with miRNA mimics were analysed after incubation for 3 weeks at soft agar format. The details are described in the Supplementary Experimental procedures, Supplementary Information.

### Cell cycle and apoptosis analysis

Cell cycle distribution was analysed using PI (Sigma-Aldrich, St, Louis, MO, USA). Caspase-3/-7 activity was assessed using a Caspase-Glo^®^ 3/7 reagent (Promega), according to the manufacturers’ protocols. Apoptotic cell death was analysed using an Annexin V-FITC conjugate and PI Apoptosis Detection Kit (BD Biosciences, San Jose, CA, USA). Analysis of cell cycle and apoptosis was performed using flow cytometry analysis on a FACS-Calibur system (BD Biosciences) and CellQuest-Pro software (BD Biosciences), according to the manufacturers’ protocols and previous description^28^. The details are summarised in the Supplementary Experimental procedures, Supplementary Information.

### Wound-healing assay

A549 cells were plated with a density of 3 × 10^5^ cells/well in six-well plates. The next day, each well was consistently scratched using the plastic pipette tip and transfected with miRNA mimics. Afterwards, cells were observed as time-dependent using a microscope (Nikon Inc., Melville, NY, USA; Eclipse 50i).

### Trans-well migration assay

Cellular migration was observed using the Trans-well system (Corning Inc., Corning, NY, USA). The inside of the Trans-well plates were coated with 0.1% gelatin (Sigma-Aldrich). The cells were transfected with miRNA mimics. After 4 h, the transfected cells were harvested and seeded at a density of 3 × 10^4^ cells/well in 0.2 ml of serum-free medium in the Trans-well top chamber, and the lower chamber was filled with 0.5 ml of medium containing 10% serum. After incubation for 48 h, the lower membrane was fixed with 95% ethanol, stained with 0.2% crystal violet (Sigma-Aldrich) for 30 min at a room temperature, washed with PBS buffer and observed using a microscope (Nikon Inc.). Data were recorded from three random fields of the lower membrane surface and data analysis was performed in triplicate for independent samples.

### Zymography analysis

Samples were collected from the medium of transfected cells, mixed with loading buffer (without reducing and heating) and loaded into a SDS-PAGE gel, which was created with 1% gelatin solution. Afterwards, the gel was washed with 2.5% Triton X-100 (Sigma-Aldrich) solution to remove the SDS and incubated in incubation buffer (50 mM Tris-HCl (pH 8.0), 10 mM CaCl_2_ and 0.02% NaN_3_) at 37 °C. After incubation for 48 h, the gel was stained and destained with a Coomassie blue (Sigma-Aldrich) developing buffer and destaining buffer, respectively. The images were recorded using a Gel Doc XRS imager (Bio-Rad).

### Animal studies

Balb/c nude mice were purchased from Orient Bio Inc. (Seongnam, Korea) and were used under the animal laboratory conditions according to the approved guidelines of the Institutional Animal Care and Use Committee of CHA University College of Medicine. Prior to the beginning of an animal experiment, mice were anaesthetised by intraperitoneal injection using a cocktail of Zoletil 50 (Virbac, Carros, France) and Rompun 2% (Bayer Korea Ltd., Seoul, Korea) as previously described^29^. Human tumour xenograft models were created by subcutaneous injection of A549 cells and then grown for 5 weeks. After 5 weeks, miRNA mimic was subcutaneously injected into the immediately surrounding the tumour with the cationic liposome Lipofectamine 2000 (Invitrogen) with reference previously described^2,30^. The size of the generated tumour was evaluated using a tumour volume calculation formula of (*W* × *W* × *L*)/2 as previously described^31^. Lung metastatic models were developed by slow injection into the tail vein of mouse with fire-fly luciferase (Fluc) stable A549 cells as previously described^2^. Bioluminescence imaging for the metastatic spread was measured using an IVIS 200 imaging system (Xenogen Corporation, Berkeley, CA, USA) after intraperitoneal injection with d-luciferin substrate (Xenogen; PerkinElmer, Waltham, MA, USA) and then quantified for the region of interest using a Living Image 3D Software (version 3.0, Xenogen; PerkinElmer) with reference previously described^32,33^. Animal studies and its ex vivo studies are described in the Supplementary Information, Supplemental Experimental procedures.

## Electronic supplementary material

Completely Revised Supplementary Information

Supplementary Figure 1

Supplementary Figure 2

Supplementary Figure 3

Supplementary Figure 4

Supplementary Figure 5

Supplementary Figure 6

Supplementary Table 1
