# Supplementary material for: The novel hsa-miR-12528 regulates tumourigenesis and metastasis through hypo-phosphorylation of AKT cascade by targeting IGF-1R in human lung cancer
Source: Cell Death Dis. 2018 May 1;9(5):493. doi: 10.1038/s41419-018-0535-8 (PMC5928042; doi:10.1038/s41419-018-0535-8)
Supplement: Supplementary file 1 — Completely Revised Supplementary Information [file 41419_2018_535_MOESM1_ESM.docx]

**1. Supplementary DATA**

**1.1. Supplementary Figure Legends**

**Supplementary Figure 1, related to Figure 2. Transfection efficiency of miRNA mimics/inhibitor for hsa-miR-12528 and the effect of hsa-miR-12528 on regulating the IGF-1R in the human-derived cell lines including A549 cells.** Transfection efficiency of miR-12528 mimic or inhibitor. During 48 h, the miR-12528 mimic was transfected in a dose-dependent manner in A549 cells and consequently optimized to 100 nM **(A)**. The efficiency of miR-12528 is indicated when each mimic was transfected at 100 nM **(B)**. The **(A)** and **(B)** data were quantified using quantitative RT-PCR and an miScript miRNA assay performed in triplicate for independent samples and normalized to RNU6B. Target validation of miR-12528 and influence of miR-12528 on regulation the IGF-1R. IGF-1R mRNA **(C)** and protein **(D)** levels in A549 cells were evaluated using qRT-PCR and Western blot analysis after 48 h post-transfection with a dose-dependent by miR-12528 mimic. To validate the targets for miR-12528, the pmirGLO-plasmid was recombined by inserting the 3'-UTR of IGF-1R (9748 to 10273; 526 bp), EGFR (3911 to 4678; 768bp), SRC (3091 to 4020; 930 bp), NRAS (3435 to 4193; 759 bp) and IKBKβ (3057 to 3852; 796 bp) mRNA **(E)**. The recombinant pmirGLO-constructs and miRNA mimics were co-transfected using lipofectamine, and subsequently luciferase activity was assessed after 48 hours post-lipofection and normalized to *Renilla* luciferase **(F)**. To evaluate the modulatory effect of the IGF-1R by miR-12528 in other human-derived cell lines; NCI-H226, -H596, -H460, SK-MES-1 (lung carcinoma), HEK293T (embryonic kidney), Hep G2 (liver hepatocellular carcinoma), MCF7 (breast carcinoma) and HeLa (cervix carcinoma), they were transfected by lipofection with either NC or miR-12528 mimic, and then IGF-1R protein levels were assessed with western blotting after 48 hours post-transfection **(G)**. The data are shown as the Bars; mean ± S.E.M; **p* <0.05 and ***p* <0.01.

**Supplementary Figure 2, related to Figure 3. FACS analysis on cell cycle and apoptosis.** Influence of miR-12528 during cell cycle. DNA-histogram plots and the numbers of DNA-distribution units for the distribution of the DNA content in A549 cells transfected with miR-12528 (100 nM), and in A549 cells of serum-starved conditions, which are a positive model for inhibition of the G1/S- phase transition **(A)**. In Figure 3C, the numerical average values of DNA-distribution units on each cell cycle phase **(B)** and on G1-phase **(C)**. Effect of miR-12528 on programmed cell death. Analysis of apoptotic cell death in A549 cells treated with a hydrogen peroxide (H_2_O_2_), which is a mediator of apoptosis. This result provides a positive model to analyse the process of apoptotic cell death **(D)**. In dot plots of Figure 3D, Graphical presentation and comparative analysis for the numerical averages of the distribution units on LR- and UR-regions **(E)**. Histogram plots and graphical presentation for the numerical averages of the Annexin V-FITC distribution units only, related to Figure 3D. Histogram plots indicate the distribution units for Annexin V-FITC fluorescence intensity conjugated at the translocated phospholipid bilayer of the plasma membrane, which is morphological feature during apoptosis **(F)**.

**Supplementary Figure 3, related to Figures 2 and 3. Knock-down effect of endogenous hsa-miR-12528 in WI-38, normal cells.** Influence on modulating the IGF-1R expression. The miRNA-target interaction was analysed using western blotting after 48 hours post-lipofection with NC, endogenous miR-12528 inhibitor (ASO-12528) or IGF-1R-specific siRNA in WI-38, normal cells (si-IGF-1R was used as a positive control in regulating the IGF-1R, target gene) **(A)**. The expression levels of pro-IGF-1R and IGF-1Rβ protein were semi-quantified by normalizing to GAPDH using a NIH image J program in independent samples **(B)**. Influence on cellular proliferation. Cellular viability in WI-38 cells was assessed in a time-dependent manner using a XTT test solution after post-transfection with either NC or ASO-12528 **(C)**. The data are shown as the Bars; mean ± S.E.M; **p* <0.05 and ***p* <0.01.

**Supplementary Figure 4, related to Figure 4. Tumourigenic xenograft models injected with A549 cells.** Subcutaneous xenograft *in vivo* models derived from A549 cells. Tumourigenesis was generated for approximately 5 weeks after injection of A549 cells in total 5 mice. After 5 weeks, the generated tumour masses were recorded or imaged and analysed using a tumour volume calculation formula of (W*W*L)/2 in both the generated right and left tumours in 5 mice **(A and B)**. The tumour tissue sections in both buttock flanks were expressed with H&E, IHC (Ki-67) staining and TUNEL labelling. The right and left tumour sections within each individual mouse show that the ratio of proliferative- and apoptotic cells was similar **(C)**.

**Supplementary Figure 5, related to Figures 1E and 2. The expression profiling and relationship between IGF-1R and hsa-miR-12528 in NSCLC-patient tissues.** The correlation between the expression of *IGF-1R* gene and miR-12528 was determined in A549 cells transfected or non-transfected (-) with 50 nM IGF-1R-sepcific siRNA, prior to stimulation with foetal bovine serum (+; 10% and -; untreated). This result shows that the miR-12528 expression increased when IGF-1R expression was silenced in A549 cells stimulated or unstimulated with the FBS **(A)**. Expression profiling in NSCLC-patient tissues. The IGF-1R or miR-12528 expression profiling was assessed in NSCLC tissues of 20 patients compared with matched normal tissues. The miR-12528 expression was measured using quantitative RT-PCR in NSCLC tissues of 20 patients and expressed as a 2^-ΔCt; delta-threshold cycle value by normalizing to RNU6B **(B)**. IGF-1R expression profiling was measured using RT-PCR and gel electrophoresis, and semi-quantified by normalizing to 18S rRNA using a NIH image J program **(C and D)**. The data are shown as the Bars; mean ± S.E.M; **p* <0.05 and ***p* <0.01.

**Supplementary Figure 6, related Figure 5. Influence of hsa-miR-12528 on lung metastatic *in vivo* models.** These data show the degree of metastatic spread for total 5 weeks in the lung metastasis mouse models that were injected into the tail vein with either NC, miR-12528, or ASO-12528 mimics-transfected Fluc-stable A549 cells. After (10 min) an intraperitoneal injection of D-luciferin substrate, bioluminescence was measured in an exposure (2 min) to Xenogen. The expressed bioluminescence activity represents the degree of metastatic spread.

**Supplementary Table 1, related to Figure 1E and Supplementary Figure 5. Information on the NSCLC patient tissue pairs.** The clinical information of 20 human lung tissue pairs

**1.2. Supplementary Experimental Procedures**

**Cell lines and Culture.** WI-38 (diploid normal lung fibroblast cell line), WI-38 VA13 (SV-40 transformed human lung epithelial-like cell line), BAE-2B (immortalized normal human lung epithelial cell line), A549, NCI-H1299, SK-LU-1 (lung adenocarcinoma), NCI-H596 (lung adenosquamous carcinoma), NCI-H226, SKMES-1 (lung squamous carcinoma), NCI-H460 (lung large-cell carcinoma), HEK293T (embryonic kidney), Hep G2 (liver hepatocellular carcinoma), MCF7 (breast carcinoma), and HeLa (cervix carcinoma) cell lines were obtained from American Type Culture Collection (ATCC, Manassas, VA, USA) and Korean Cell Line Bank (KCLB, Seoul, Korea). The WI-38 VA13, A549, NCI-H1299, NCI-H596, NCI-H226, NCI-H460, Hep G2, MCF7 and HeLa cell lines were maintained in RPMI-1640 (Welgene, Deagu, Korea). SK-LU-1, SKMES-1 and HEK293T cell lines were maintained in DMEM (Welgene). WI-38 cell line was maintained in EMEM (Welgene). BAE-2B cell line was maintained in DMEM/F12 (Gibco, Grand Island, NY). All cell lines were supplemented with 10% FBS (Welgene), 1% penicillin (100 U/mℓ, Welgene) and streptomycin (100 µg/mℓ, Welgene) mixture. These cell lines were incubated at 37°C with 5% CO_2_. The firefly luciferase (Fluc) stable A549 cell line was integrated randomly into the host cellular genome by transiently transfection using Lipofectamine 2000 reagent (Invitrogen, Carlsbad, CA, USA) with the CMV promoter based pGL 4.51-vector (Promega, Madison, WI, USA). Fluc A549 stable cell line was selected with G418 (800 µg/ml, Gibco) for 2 weeks in transfected single conditional cells and collected via picking of a single colony after 2 weeks. Culture medium containing G418 was replaced at regular intervals. Collected colonies were transferred into 6-well plates and subsequently maintained in 100 mm plates via subculture. Stable Fluc expression was assessed via Fluc activity for D-luciferin reaction at luminometer (BioTeK Inc., Winooski, VT, USA). The selected cell line was maintained in RPMI-1640 containing 10% FBS, 1% P/S and 800 µg/ml G418. The detailed procedures are referenced in the IVIS imaging protocols (item: Stable Transfection of Adherent Cells) provided by PerkinElmer, Inc..

**RNA isolation and cDNA synthesis.** Total RNA was isolated using the TRIzol reagent (Qiagen, Hilden, Germany), according to the manufacturers’ protocol. After a 75% ethanol wash, the RNA was dissolved in 0.1% DEPC-treated water (Sigma-Aldrich, St, Louis, MO, USA). Tissue fractions were homogenized in TRIzol reagent (Qiagen, Hilden, Germany; 50 to 100 mg tissue fraction per 1 mℓ). The cDNA for gene expression was synthesized using a reverse transcription Kit (Invitrogen, Carlsbad, CA, USA) and cDNA for miRNA expression was synthesized using the miScript II RT Kit (Qiagen), according to the manufacturers’ protocol.

**MiRNA mimic design and Transfection.** miRNA mimics were purchased and designed from Genolution pharmaceuticals Inc. (Seoul, Korea). NC is scrambled RNA; non-homologous from human genome sequence. The si-IGF1R is an siRNA for the IGF-1R genes and was used as a positive control. The sequence information and list of miRNA mimic were recorded in the table below. Transfection was performed using a Lipofectamine 2000 Reagent (Invitrogen), according to manufacturers’ protocol. The media containing the mixture of transfection reagents was removed after 4 to 6 h of transfection and then changed to fresh culture media without antibiotics. The transfected cells were harvested after 48 h post-transfection and then were analysed in the majority of experiments. The miR-12528 transfection efficiency was optimized through dose-dependent transfection and quantitative real-time PCR (qRT-PCR) analysis. Experiments were performed using the optimum concentration of 100 nM (Figures S1A, B).

| No | miRNA/siRNA | Position | Sequence (5´ - 3´) |
| --- | --- | --- | --- |
| 1 | Si-Dicer | Sense | UGCUUGAAGCAGCUCUGGAUU |
|  |  | Antisense | UCCAGAGCUGCUUCAAGCAUU |
| 2 | Negative Control; NC | Sense | ACGUGACACGUUCGGAGAAUU |
|  |  | Antisense | UUCUCCGAACGUGUCACGUUU |
| 3 | si-IGF-1R | Sense | GCAAUUUGCUCAUUAACAUUU |
|  |  | Antisense | AUGUUAAUGAGCAAAUUGCUU |
| 4 | Novel hsa-miR-12528 | Sense | CGGAAUGGGGGGGGGGGCCG |
|  |  | Antisense | CGGCCCCCCCCCCCAUUCCG |
| 5 | ASO-12528 | Antisense | Complementary sequence of miR-12528 sense  ASO, Antisense Oligonucleotide |

**miScript miRNA assay and qRT-PCR analysis.** The miRNA or gene expression was analysed using the qRT-PCR analysis (Bio-Rad, Hercules, CA, USA). The used primer sequences were designed by Bioneer, Inc. (Daejeon, Korea) and noted in the table below. The miScript universal primer used in miRNA assay was purchased from Qiagen, Inc. The qRT-PCR analysis was performed using a SYBR Green PCR Kit (Qiagen) and Real-Time PCR Detection System (Bio-Rad), according to manufacturers’ manual. RNU6B (miScript Primer Assay) and 18S rRNA were used as internal controls to normalize the target genes or miRNAs, and the results are expressed as 2(-delta (delta) threshold cycle); 2^-∆∆(C)T^ values.

| Name | Genbank Accession | Zone | Primer sequence (5´-3´) |
| --- | --- | --- | --- |
| miR-21 | NR_029493 | Forward | TAGCTTATCAGACTGATGTTGA |
|  |  | Reverse | miScript universal primer |
| let-7a | NR_029476 | Forward | TGAGGTAGTAGGTTGTATAGTT |
|  |  | Reverse | miScript universal primer |
| miR-12528 | Novel | Forward | CGGAATGGGGGGGGGGGCCG |
|  |  | Reverse | miScript universal primer |
| RNU6B | NG_034215 | Forward | CTGCGCAAGGATGACACG |
|  |  | Reverse | miScript universal primer |
| IGF-1R | NG_009492 | Forward | TCAGGACGGCTACCTTTACC |
|  |  | Reverse | CTTGGGGTTCTCTGTGACCT |
| Dicer | NG_016311 | Forward | CAAGTGTCAGCTGTCAGAACTC |
|  |  | Reverse | CAATCCACCACAATCTCACATG |
| 18S rRNA | K03432 | Forward | TACCTACCTGGTTGATCCTG |
|  |  | Reverse | GGGTTGGTTTTGATCTGATA |

**Western blot analysis.** Proteins were extracted using a PRO-PREP™ (iNtRON, Seoungnam, Korea) lysis buffer that was supplemented with phosphatase inhibitor cocktail (Thermo Scientific, Rockford, IL, USA), separated on SDS-PAGE gels and transferred to polyvinylidene fluoride membranes (pore size, 0.45 µm, GE Healthcare Life Sciences, Piscataway, NJ, USA). The membranes were blocked for 1h in 5% skim milk (BD Biosciences, San Jose, CA, USA) and subjected to immunoblotting analysis using primary and secondary antibodies. All primary antibodies were incubated overnight at 4°C, and then secondary antibodies were incubated for 2 to 3 h at a room temperature. The membrane was washed with TBS-T buffer and the signal was detected using West-Q Pico ECL solution (GenDEPOT, Barker, TX, USA) and a LAS-4000 imager (FUJIFILM Medical Systems, Woodbridge, CT, USA). GAPDH was used as an internal control. Primary and secondary antibodies were purchased at Santa Cruz Biotechnology (Santa Cruz, CA, USA) and Cell Signaling Technology (CST, Inc., Danvers, MA, USA), and the information is shown in the table below. Antibodies were diluted at a ratio of 1:1,000 in 1% BSA (Bioworld Technology, Minneapolis, MN, USA). Semi-quantification analysis was performed by triplicate repetition in independent samples and using a NIH ImageJ program.

| Form | Antibody | Company | Cat. No |
| --- | --- | --- | --- |
| Primary | IGF-1Rβ (C-20) | Santa Cruz | SC-713 |
| Primary | Akt1 (B-1) | Santa Cruz | SC-5298 |
| Primary | p-Akt1 (Thr 308) | Santa Cruz | SC-135650 |
| Primary | mTOR (Ser 2481) | Cell Signaling | #2972 |
| Primary | p-mTOR (Ser 2448) | Santa Cruz | SC-101738 |
| Primary | XIAP | Cell Signaling | #2042 |
| Primary | Bcl-2 (C-2) | Santa Cruz | SC-7382 |
| Primary | α-Tubulin (B-7) | Santa Cruz | SC-5286 |
| Primary | Cdk4 (C-22) | Santa Cruz | SC-260 |
| Primary | Cdk2 (D-12) | Santa Cruz | SC-6248 |
| Primary | Rb (C-2) | Santa Cruz | SC-74562 |
| Primary | p-Rb (Thr 821/826) | Santa Cruz | SC-16669 |
| Primary | GAPDH (FL-335) | Santa Cruz | SC-25778 |
| Secondary | Goat anti-Rabbit IgG-HRP | Santa Cruz | SC-2004 |
| Secondary | Goat anti-Mouse IgG-HRP | Santa Cruz | SC-2005 |
| Secondary | Donkey anti-Goat IgG-HRP | Santa Cruz | SC-2020 |

**Proliferation assay.** In short-term proliferation assay, A549 or WI-38 cells were plated at a density of 5 x 10^3^ cells per well in 96-well plates. The next day, each well was transfected with miRNA mimics and assessed at 0 and then at 24h intervals for 3 or 4 days. Proliferation rates were assessed using a XTT test solution, which was prepared by mixing 5 mℓ of XTT labelling reagent with 100 μℓ of electron-coupling reagent (XTT, Roche, Mannheim, Germany). Absorbance was measured at a wavelength of 450 nm and 690 nm (background) using VICTOR^3^ (PerkinElmer Inc.). The experiment was performed in triplicate for independent samples.

**Colony formation assay.** In colony-forming assay, pre-coating was equally pre-prepared in 2 mℓ volume of RPMI-1640 medium contained with 0.5% agarose to each 60-mm dishes. A549 cells were plated with a density of 3 x 10^5^ cells per well in 6-well plates. The next day, each well was transfected with miRNA mimics. After 4 h, the cells were harvested and then counted in hemocytometer. Consecutively, the cells (7 x 10^3^) were gently mixed in the final 2 mℓ of RPMI-1640 medium supplemented with 0.3% agarose and then were layered on the pre-coated dishes with base agarose. After 3-weeks, cell-derived colonies were stained with 0.2% Crystal Violet (Sigma-Aldrich) and counted using a dissecting microscope. The experiment was repeated in triplicate for independent samples.

**Active Caspase 3/7 Assay.** A549 cells were plated at a density of 1 x 10^4^ cells per well in a white-walled 96-well cell culture plate (Greiner Bio-One, Kyunggi-do, Korea). The next day, each well was transfected with miRNA mimics and then incubated at 37°C with 5% CO_2_ for 48 h. Caspase-Glo ® 3/7 reagent (Promega) was added to each well containing blank, untransfected cells and transfected cells in culture medium, and then incubated at room temperature for 3 h, according to the manufacturers’ protocol. The luminescence was assessed at luminometer (BioTeK Inc., Winooski, VT, USA). The experiment was repeated in 9 times for independent samples.

**Cell cycle and Apoptosis analysis.** In the distribution of cell cycle, A549 cells were plated at a density of 1x10^5^ cells per well in 6-well plates and transfected with miRNA mimics the next day. After 48 h, transfected cells were harvested, fixed for 1 h to overnight in 70% ice-cold ethanol, washed with cold 1x PBS buffer and treated with Triton X-100 (Sigma-Aldrich) and RNase A (Elpis, Daejeon, Korea) for 30 min at RT. Permeabilized cells were stained with 50 µg/ mℓ propidium iodide (PI, Sigma-Aldrich). Apoptotic cell analysis was performed using an Annexin V-FITC conjugate and PI Apoptosis Detection Kit (BD Biosciences, San Jose, CA, USA). Transfected cells were resuspended in 1x Annexin V binding buffer and then stained using an Annexin V-FITC/PI solution, according to the manufacturers’ protocol. The PI fluorescence events of 10,000 cells in cell cycle analysis, and a total of 10,000 cells in apoptotic cell death analysis were analysed via gating tools from each sample. DNA content analysis or an apoptotic cell assay was performed using flow cytometry analysis on a FACS-Calibur system (BD Biosciences) and CellQuest-Pro software (BD Biosciences), according to the manufacturers’ protocols.

**Subcutaneous tumour xenograft model.** A total of 26 tumourigenic mice models were used, where 21 mice (Male Balb/c nude, 6 weeks old, Orient Bio, Seoungnam, Korea) were divided into 7 mice per group. All mice were injected subcutaneously (s.c) with each 3 x 10^6^ A549 cells in both buttock flanks and stabilized for 5 weeks. After 5 Weeks, the PBS (Mock), NC, miR-12528 and ASO-12528 mimics were subcutaneously injected in the segment immediately adjacent to the tumour with the cationic liposome Lipofectamine 2000 mixture twice weekly for 4 weeks (400 pmol per 1 injection, A Group; Left; Mock-Right; NC, B Group; L; Mock-R; miR-12528 and C Group; L; Mock-R; ASO-12528). After a total of 4 weeks from the time of mimic injection, 7 mice per group (a total of 21 n) were sacrificed and the tumour was excised for *ex vivo* analysis. Tumour mass was measured using a caliper position of width (W) and length (L), and tumour volume was analysed using a tumour volume calculation formula of (W*W*L)/2. Additionally, tumour tissues were embedded in paraffin. Tumour sections were stained with haematoxylin and eosin (H&E).

**Immunostaining and TUNEL assay.** Tumour tissues were fixed with 4% paraformaldehyde; PFA, embedded in paraffin and sectioned (5 µm). Immunostaining was performed on paraffin sections and mounted on slides. Paraffin sections were stained with H&E and labelled using an anti-Ki67 antibody (Santa Cruz; SC-15402) for immunohistochemistry. Primary antibody; Ki67 was used at a ratio of 1:200 and incubated overnight. Subsequently, sections were incubated for 1 to 2 h using a horseradish peroxidase-conjugated (HRP) secondary antibody (Dako, Seoul, Korea; K4003). Visualization or validation was performed using a 3,3´-diaminobenzidine kit (Thermo Scientific; TA-125-HDX), and haematoxylin was used as counterstain in IHC staining. In TUNEL assay, Tumour tissues of sacrificed mice were fixed with 4% PFA and then embedded in paraffin. Paraffin sections were deparaffinized in water and placed in 3% H_2_O_2_ for 10 min at RT to quench endogenous peroxidase. TUNEL assay was performed using a TUNEL Apoptosis Detection Kit (Upstate Biotechnology, Charlottesville, VA, USA), according to the manufacturers’ instructions. Images were displayed at randomly position using a microscope (Nikon Inc., Melville, NY, USA; Eclipse 50i) and i-solution image analyser (Daejeon, Korea). Positive results for Ki67 and TUNEL were indicated by brown nuclear staining.

**Metastatic *in vivo* model and bioluminescence imaging (BLI).** A metastatic *in vivo* model was generated in a total of 24 mice (Orient Bio, male Balb/c nude, 6 weeks old) that were divided into NC, miR-12528 and ASO-12528 groups. Each group comprised 8 mice. Mice were anesthetized by intraperitoneal injection using a cocktail; 3:1 ratio of Zoletil 50 (Virbac, Carros, France) and Rompun 2% (Bayer Korea Ltd., Seoul, Korea). The A549_CMV_Fluc stable cell lines were overexpressed with 100 nM-target miRNA mimics. After post-transfection for approximately 6 h, the transfected A549_Fluc cells were harvested and re-suspended in 1x PBS. For the real-time monitoring of metastatic spread, the transfected A549_CMV_Fluc stable cells were slowly injected into the tail vein of each mouse with 1 x 10^6^ / 1 x PBS, 100 µl per mouse. After 5 weeks, the mice were injected with 100 µℓ (150 mg/kg) of D-luciferin substrate (Xenogen; PerkinElmer, Waltham, MA, USA) by intraperitoneal injection, and then BLI was measured *in vivo* and/or using resected lung tissues 10 min later with an IVIS 200 imaging system (Xenogen Corporation, Berkeley, CA, USA). The exposure time was 2 min. Fluc activity was quantified for the region of interest (ROI), and analysed using a Living Image 3D Software (version 3.0, Xenogen; PerkinElmer). BLI analysis was performed with reference to the IVIS imaging protocols (item: Cell Preparation and Imaging Protocol) provided by PerkinElmer Inc..

**Statistical analyses.** All data results are expressed as the mean with SEM and analysed for significance in a paired t-test using the SPSS program and GraphPad Prism 5. Additionally, *p-values* ≤0.05 were considered significant.
