# Supplementary figures and images for: The novel hsa-miR-12528 regulates tumourigenesis and metastasis through hypo-phosphorylation of AKT cascade by targeting IGF-1R in human lung cancer
Source: Cell Death Dis. 2018 May 1;9(5):493. doi: 10.1038/s41419-018-0535-8 (PMC5928042; doi:10.1038/s41419-018-0535-8)

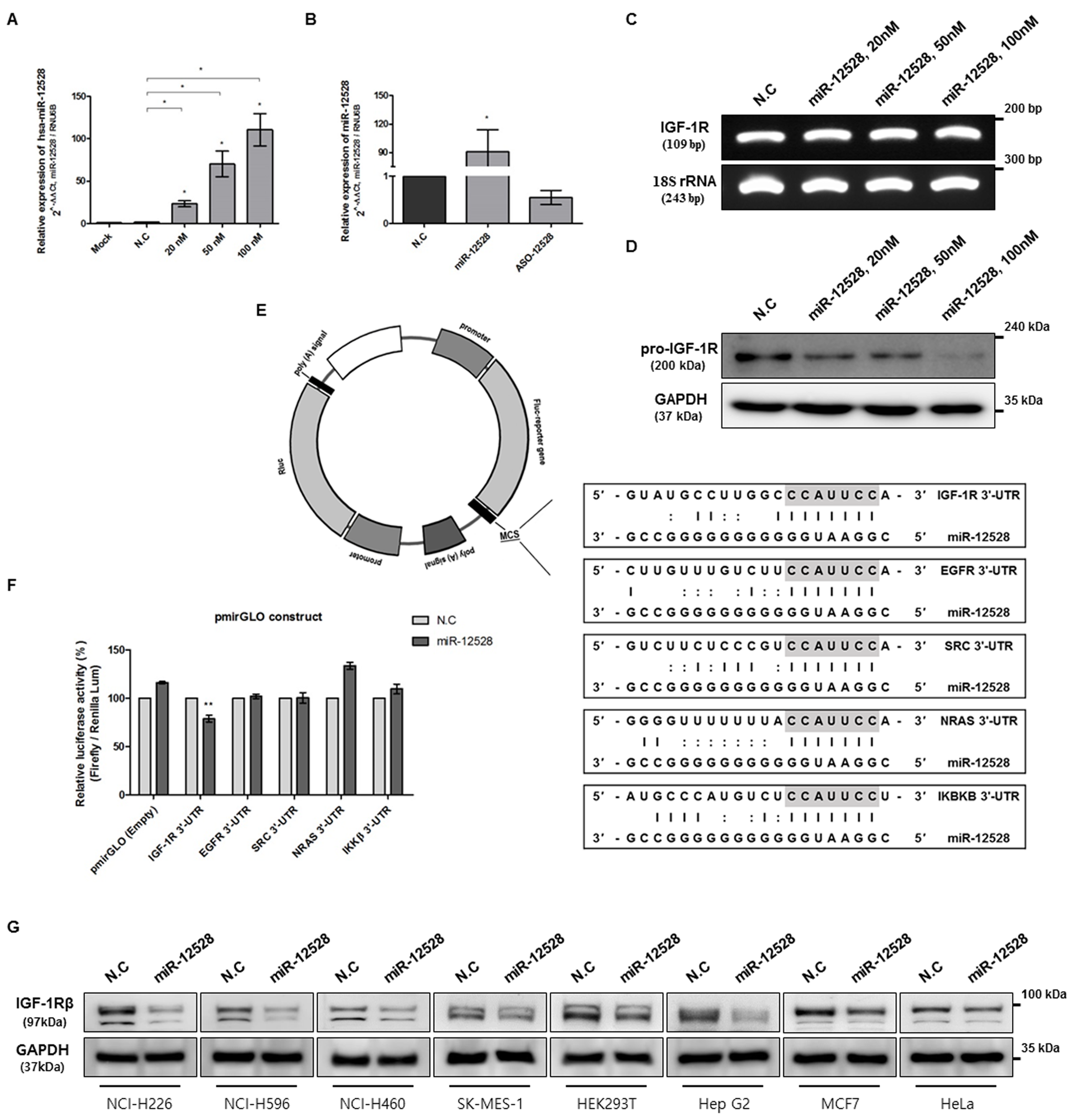

Supplement: Supplementary file 2 — Supplementary Figure 1 [file 41419_2018_535_MOESM2_ESM.tif]

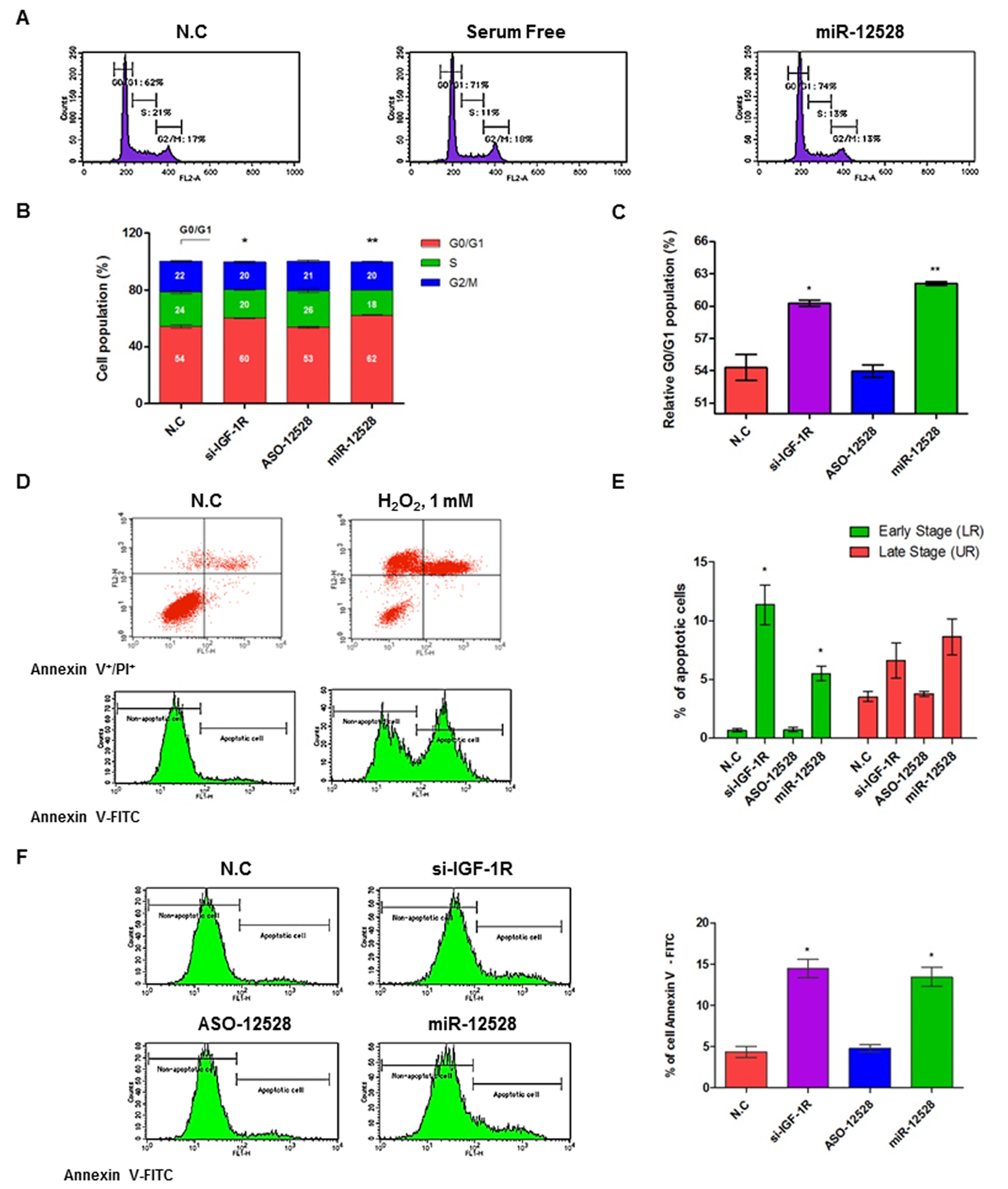

Supplement: Supplementary file 3 — Supplementary Figure 2 [file 41419_2018_535_MOESM3_ESM.tif]

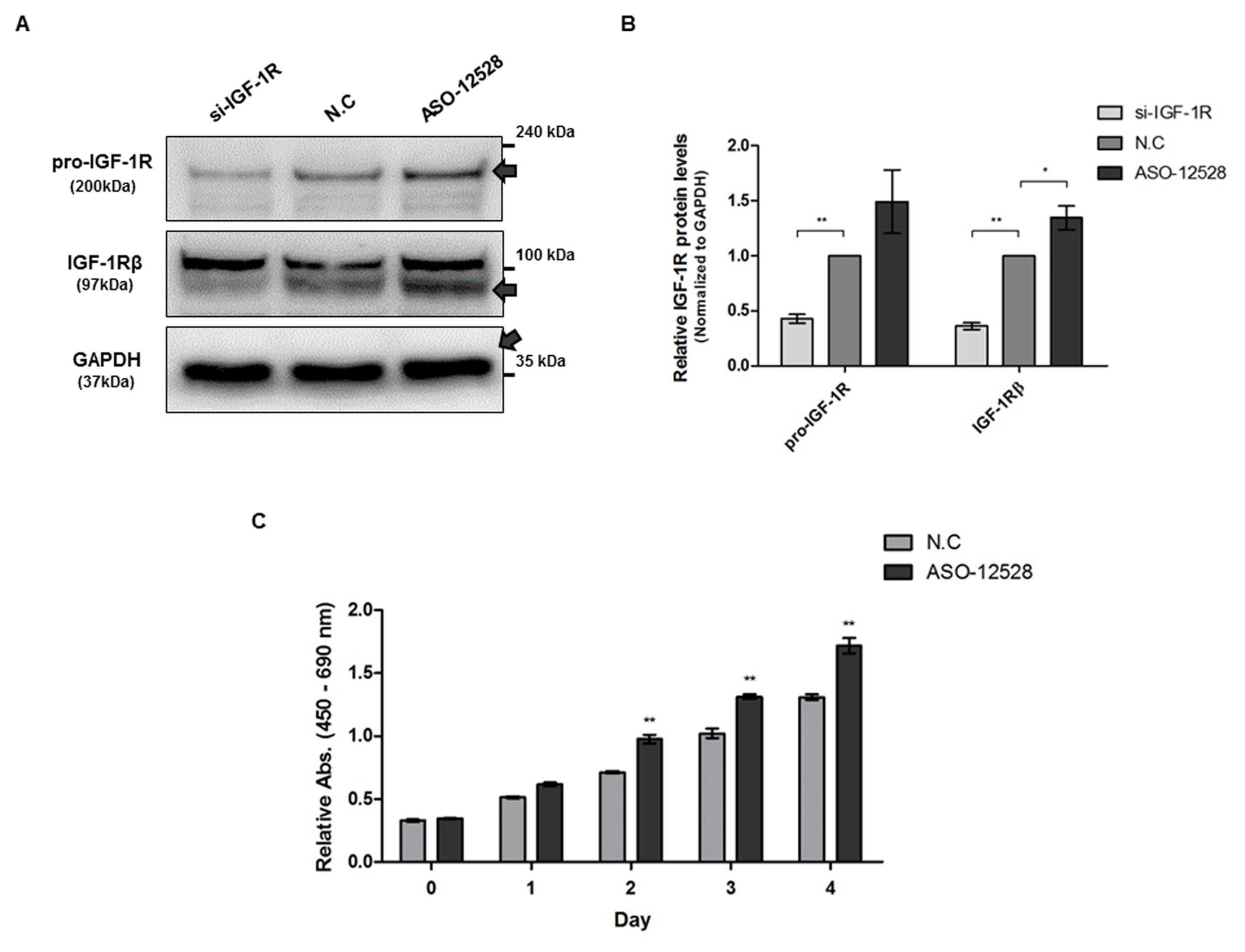

Supplement: Supplementary file 4 — Supplementary Figure 3 [file 41419_2018_535_MOESM4_ESM.tif]

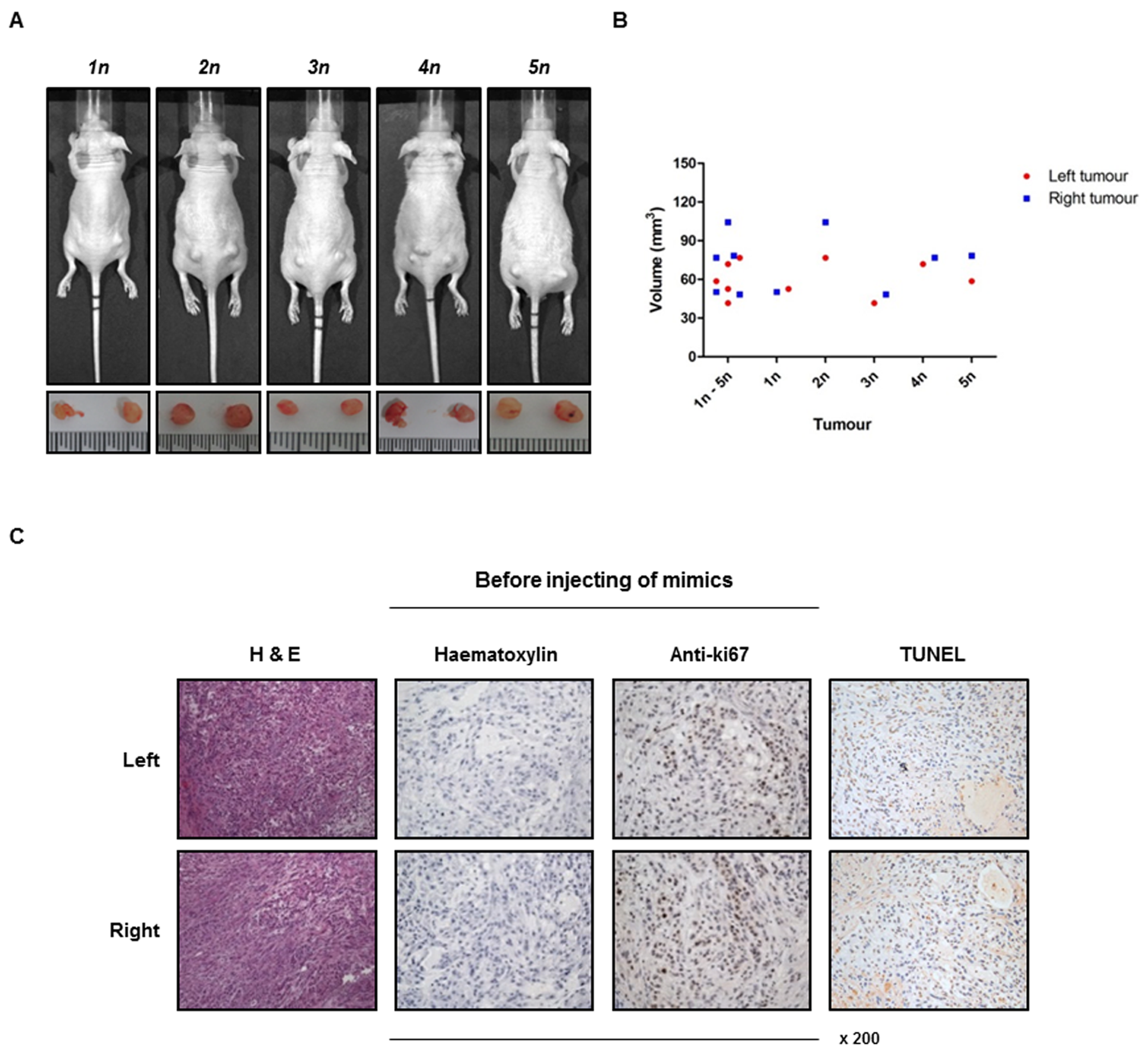

Supplement: Supplementary file 5 — Supplementary Figure 4 [file 41419_2018_535_MOESM5_ESM.tif]

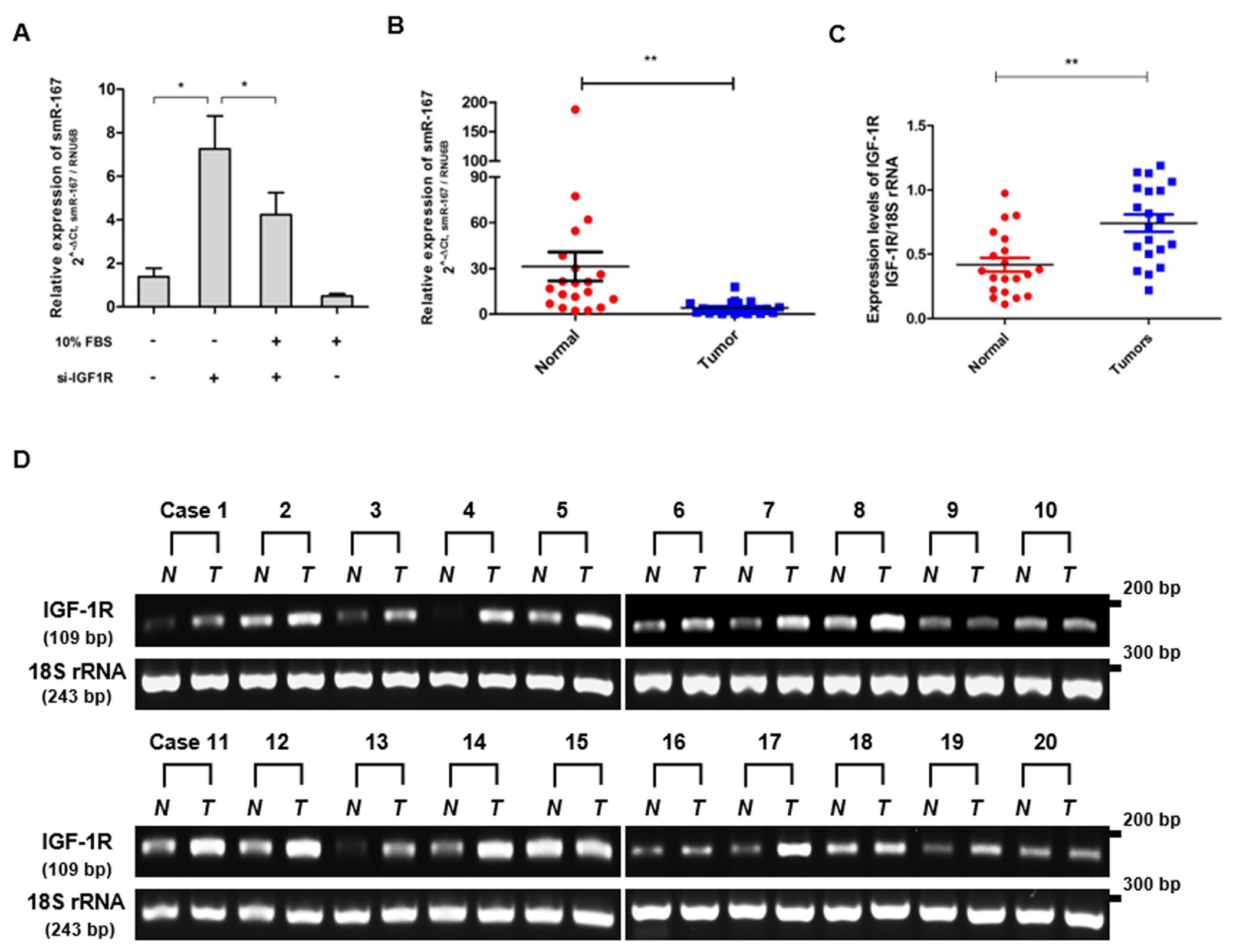

Supplement: Supplementary file 6 — Supplementary Figure 5 [file 41419_2018_535_MOESM6_ESM.tif]

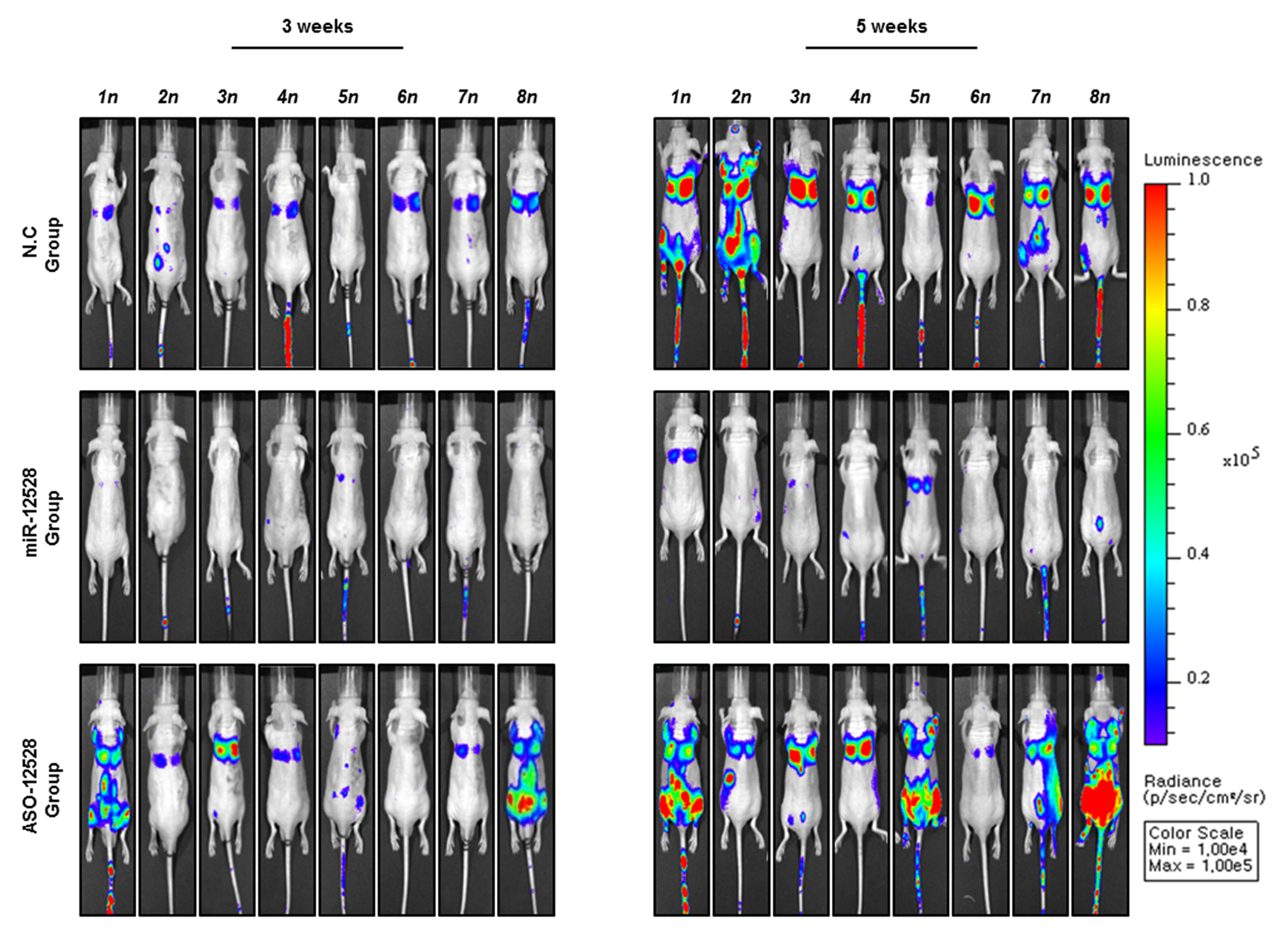

Supplement: Supplementary file 7 — Supplementary Figure 6 [file 41419_2018_535_MOESM7_ESM.tif]

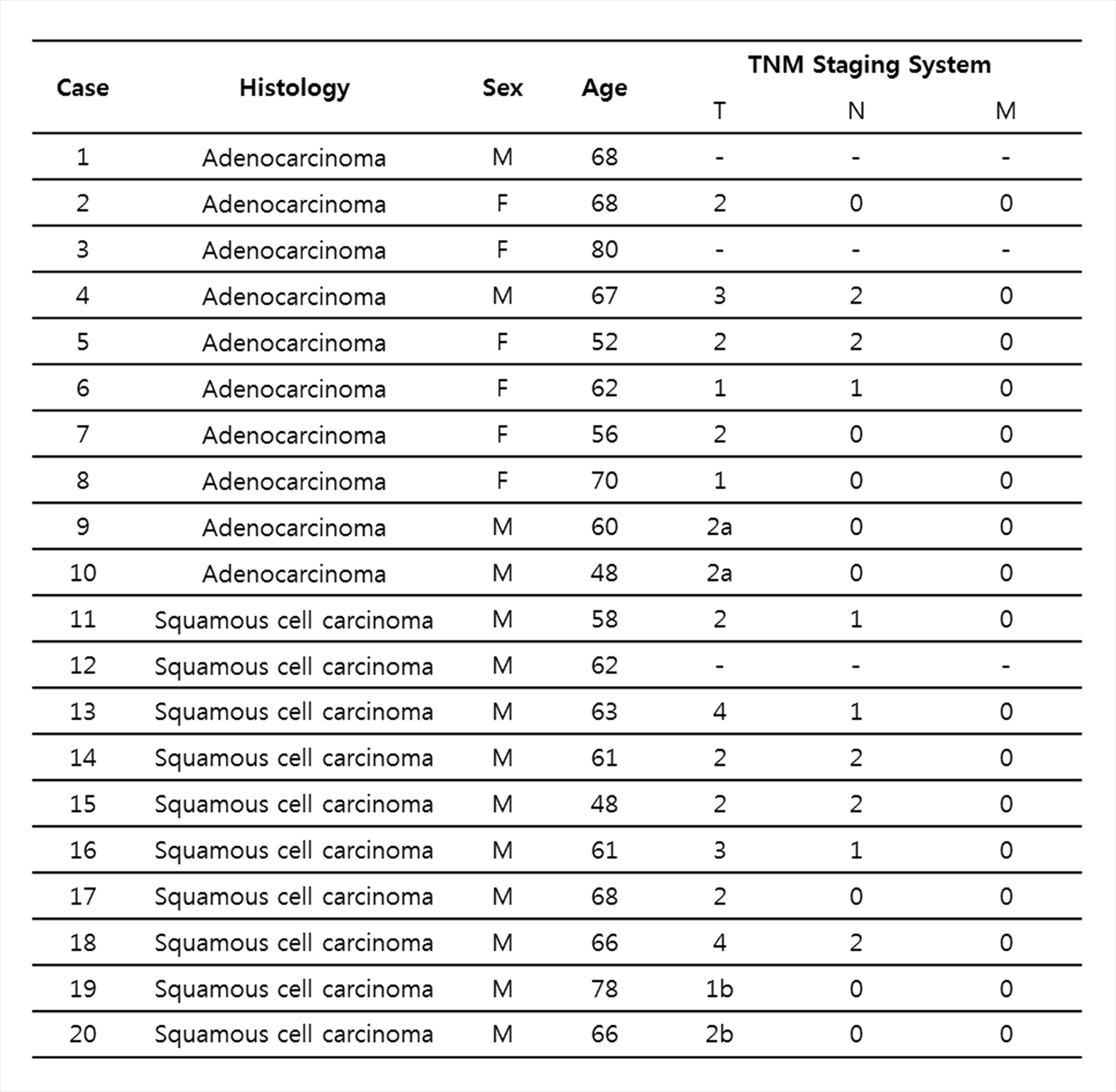

Supplement: Supplementary file 8 — Supplementary Table 1 [file 41419_2018_535_MOESM8_ESM.tif]
